# Impaired body-centred sensorimotor transformations in congenitally deaf people

**DOI:** 10.1093/braincomms/fcac148

**Published:** 2022-06-07

**Authors:** Hui Li, Li Song, Pengfei Wang, Peter H Weiss, Gereon R Fink, Xiaolin Zhou, Qi Chen

**Affiliations:** Cognitive Neuroscience, Institute of Neuroscience and Medicine (INM-3), Research Centre Jülich, Wilhelm-Johnen-Strasse, 52428 Jülich, Germany; Key Laboratory of Brain, Cognition and Education Sciences, Ministry of Education, Guangzhou, China; School of Psychology, Center for Studies of Psychological Application, and Guangdong Key Laboratory of Mental Health and Cognitive Science, South China Normal University, Guangzhou, China; Key Laboratory of Brain, Cognition and Education Sciences, Ministry of Education, Guangzhou, China; School of Psychology, Center for Studies of Psychological Application, and Guangdong Key Laboratory of Mental Health and Cognitive Science, South China Normal University, Guangzhou, China; Key Laboratory of Brain, Cognition and Education Sciences, Ministry of Education, Guangzhou, China; School of Psychology, Center for Studies of Psychological Application, and Guangdong Key Laboratory of Mental Health and Cognitive Science, South China Normal University, Guangzhou, China; Cognitive Neuroscience, Institute of Neuroscience and Medicine (INM-3), Research Centre Jülich, Wilhelm-Johnen-Strasse, 52428 Jülich, Germany; Department of Neurology, University Hospital Cologne, Cologne University, 509737 Cologne, Germany; Cognitive Neuroscience, Institute of Neuroscience and Medicine (INM-3), Research Centre Jülich, Wilhelm-Johnen-Strasse, 52428 Jülich, Germany; Department of Neurology, University Hospital Cologne, Cologne University, 509737 Cologne, Germany; Shanghai Key Laboratory of Mental Health and Psychological Crisis Intervention, School of Psychology and Cognitive Science, East China Normal University, 200062 Shanghai, China; Cognitive Neuroscience, Institute of Neuroscience and Medicine (INM-3), Research Centre Jülich, Wilhelm-Johnen-Strasse, 52428 Jülich, Germany; Key Laboratory of Brain, Cognition and Education Sciences, Ministry of Education, Guangzhou, China; School of Psychology, Center for Studies of Psychological Application, and Guangdong Key Laboratory of Mental Health and Cognitive Science, South China Normal University, Guangzhou, China

**Keywords:** congenital deafness, egocentric reference frame, dorsal attention network, frontoparietal network, default-mode network

## Abstract

Congenital deafness modifies an individual’s daily interaction with the environment and alters the fundamental perception of the external world. How congenital deafness shapes the interface between the internal and external worlds remains poorly understood. To interact efficiently with the external world, visuospatial representations of external target objects need to be effectively transformed into sensorimotor representations with reference to the body. Here, we tested the hypothesis that egocentric body-centred sensorimotor transformation is impaired in congenital deafness. Consistent with this hypothesis, we found that congenital deafness induced impairments in egocentric judgements, associating the external objects with the internal body. These impairments were due to deficient body-centred sensorimotor transformation *per se*, rather than the reduced fidelity of the visuospatial representations of the egocentric positions. At the neural level, we first replicated the previously well-documented critical involvement of the frontoparietal network in egocentric processing, in both congenitally deaf participants and hearing controls. However, both the strength of neural activity and the intra-network connectivity within the frontoparietal network alone could not account for egocentric performance variance. Instead, the inter-network connectivity between the task-positive frontoparietal network and the task-negative default-mode network was significantly correlated with egocentric performance: the more cross-talking between them, the worse the egocentric judgement. Accordingly, the impaired egocentric performance in the deaf group was related to increased inter-network connectivity between the frontoparietal network and the default-mode network and decreased intra-network connectivity within the default-mode network. The altered neural network dynamics in congenital deafness were observed for both evoked neural activity during egocentric processing and intrinsic neural activity during rest. Our findings thus not only demonstrate the optimal network configurations between the task-positive and -negative neural networks underlying coherent body-centred sensorimotor transformations but also unravel a critical cause (i.e. impaired body-centred sensorimotor transformation) of a variety of hitherto unexplained difficulties in sensory-guided movements the deaf population experiences in their daily life.

## Teaser

Li *et al*. investigate the cognitive and neural mechanisms underlying the potentially altered spatial reference frame system in deaf people and reveal that early auditory deprivation impairs egocentric processing, which is associated with an abnormally increased cross-talk between task-positive and task-negative networks, for both evoked and intrinsic neural activity.

## Graphical Abstract

**Graphical abstract:**
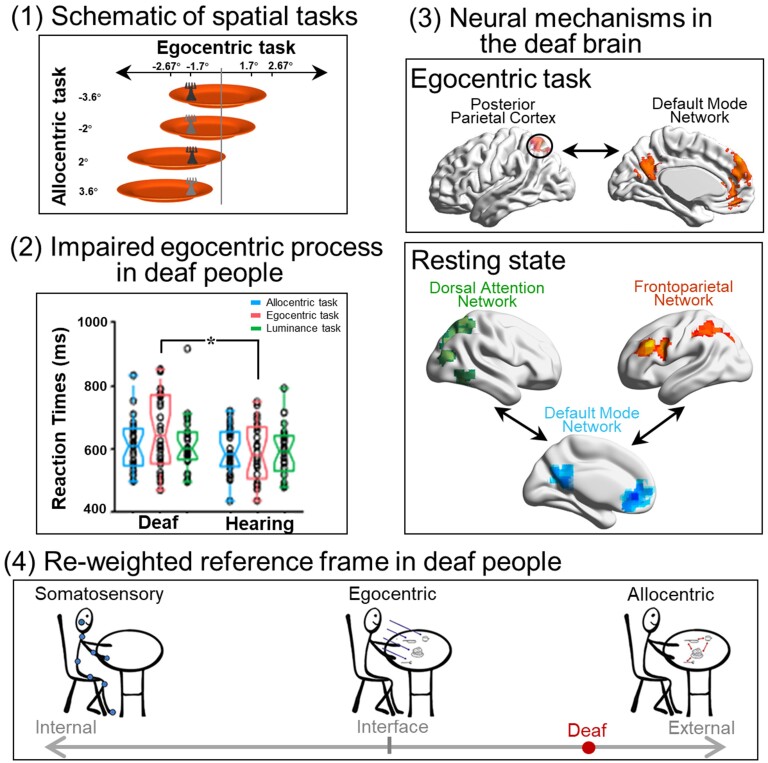

## Introduction

**Figure 1:**
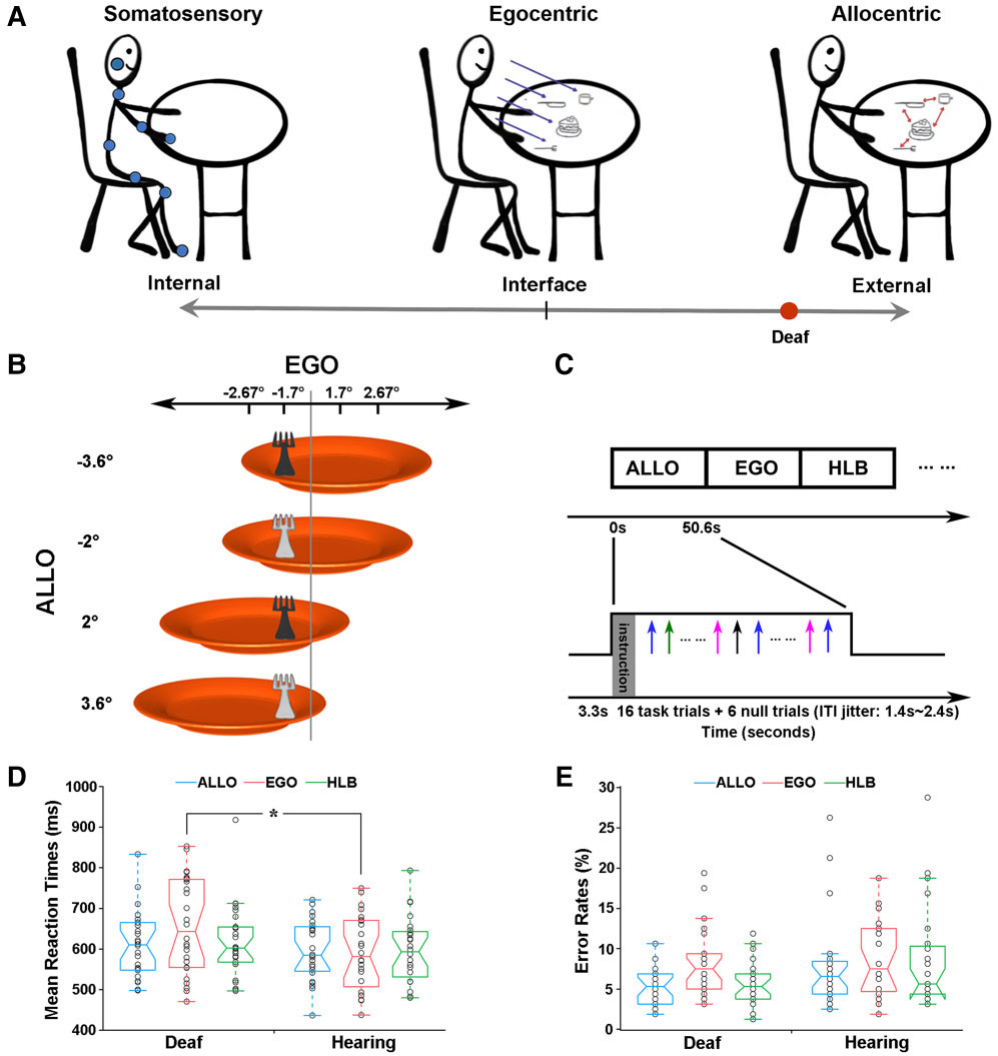
**Hypothesis, experimental stimuli, paradigm and behavioural data.** (**A**) The present hypothetical model on how the spatial reference system is biased towards the external end of the internal–external continuum by the early auditory deprivation. (**B**) Stimuli. The behavioural target is a fork lying on an orange plate. The luminance of the fork could be either light or dark grey. Four egocentric positions (−2.67°, −1.7°, 1.7° and 2.67°) and four allocentric positions (−3.6°, −2°, 2° and 3.6°) were orthogonally crossed. (**C**) Experimental paradigm and design. Three types of task blocks (ALLO, EGO and HLB) were randomly alternated without any rest blocks. Each block started with a 3.3s’ instruction. An event-related design was embedded within each block: experimental trials were intermixed with null trials (only a default blank screen) and the ITIs were adequately jittered. (**D**) Mean RTs and (**E**) error rates are shown as a function of the three types of tasks in each group, just for demonstration purposes without further statistical analyses. The asterisk indicates a statistically significant between-group difference in the GLMM analysis, *P* < 0.05.

The brain continuously generates adaptive action plans to interact effectively with the external environment. These action plans are based on the spatial representations of behaviourally relevant objects in multiple ‘spatial reference frames’, e.g. the frame with reference to our own body/body effectors (egocentric) or with reference to objects in the external environment (allocentric).^1–7^ In the typically developed brain, allocentric and egocentric processing depends on partially overlapping but distinct brain networks.^8–12^ Both spatial reference frames involve the dorsal attention network (DAN), which commonly codes the visuospatial representations underlying both frames of reference.^9,10^ The egocentric reference frame specifically involves the frontoparietal network (FPN), which supports the body-centred sensorimotor representations underlying sensory-guided actions.^13,14^ The allocentric reference frame specifically involves the medial temporal lobe (MTL) structures, which support object-/world-centred representations underlying spatial navigation.^15–17^ From a task demand point of view, the allocentric reference frame befalls at the relatively external end of the spatial reference frame’s external–internal continuum (Fig. 1A). In contrast, the anatomically anchored somatosensory/tactile reference frame occupies the relatively internal end. Interestingly, the egocentric reference frame represents the primary interface between the body and the environment by associating external objects with one’s own body^18^ (Fig. 1A).

The perception of the environment is directly related to the ability to act upon it.^4^ Early auditory deprivation modifies an individual’s interaction with the surrounding environment and fundamentally alters the perception of the external world’s.^19,20^ The fact that deafness leads to significant alterations in attentional and sensory processing of the external world is well documented (for reviews^21,22^). In sharp contrast, how the interface between the external world and the internal body is shaped by the lack of early auditory experience remains poorly understood. Auditory input plays an essential role in the normal development of body-related processes in the hearing population, such as somatosensory, motor, posture and balance processing, with all these processes closely related to the body schema for action.^23–28^ Accordingly, the lack of auditory experience in the deaf population can have a dramatic impact on these body-related processes.^19,27,29–38^ An intact vision allows an individual to use the ‘parallel input’ attributes of the visual system to simultaneously capture spatial relationships between multiple external objects and their contextual background (i.e. allocentric spatial relationships).^39^ Therefore, the intact visual system in deaf people might boost allocentric reference frame, while the use of egocentric reference frame is reduced due to deficits in the body-related processes caused by congenital deafness. Accordingly, it is reasonable to assume that the lack of early auditory input biases the spatial reference frames in the deaf brain towards the outer end of the internal–external continuum (i.e. more reliance on the allocentric reference frame) (Fig. 1A).

Using neuropsychological tests and functional MRI (fMRI) in a sample containing both congenitally deaf participants and normal hearing, typically developed controls, we thus investigated the cognitive and neural mechanisms underlying the potentially altered spatial reference frame system in deaf individuals. Based on the hypothetical model presented (Fig. 1A), we predicted that, if the lack of early auditory input impairs the body-centred egocentric reference frame and renders the spatial reference frame system more reliant on the external world-centred allocentric reference frame, then congenitally deaf participants would show worse egocentric performance while having an allocentric performance comparable to or better than normal-hearing controls. At the neural level, we aimed to investigate the altered neural network dynamics underlying the putatively impaired egocentric reference frame in congenitally deaf participants when compared with normal-hearing controls. Specifically, the egocentric reference frame could be impaired either due to (i) incoherent interaction within and/or between the task-positive networks (DAN and FPN) or (ii) hyper-cross-talk between the task-positive networks and the task-negative default-mode network (DMN). Both disturbances would result in a deficient transformation of the visuospatial representations in the DAN into the sensorimotor representations in the FPN.

## Materials and methods

### Participants

Twenty-six early deaf participants, who were undergraduate students from the School of Special Education, Beijing Union University, China, and 24 normal-hearing controls, who were undergraduate students at several other universities in Beijing, were recruited for this study. The appropriate sample size was calculated based on the G*Power toolbox.^40,41^ According to previous research, a medium effect size (*f* = 0.25) was used.^42^ With *α* error = 0.05 and power = 0.8,^43–45^ the appropriate sample size was calculated to be 22. Thus, the sample size that we adopted in the current study (26 deaf participants and 24 normal-hearing participants) should be appropriate to address our research question.

The two groups did not differ significantly in age, gender distribution and years of education (all *P*s >0.5) (Table 1). Based on deaf and normal-hearing participants’ self-reports, no participant had received a clinical diagnosis of any balance problems (vestibular dysfunction). The hearing thresholds of all deaf participants in both ears were >90 dB, which fulfils the criterion for profound deafness.^46^ The mean hearing thresholds were measured via a standard pure tone audiometry at 0.5, 1, 2 and 4 kHz. All the deaf participants became profoundly deaf at birth, and the causes of deafness were genetic (hereditary deafness) or pregnancy-related (such as maternal disease or drug side effects) (Supplementary Table 1). All the deaf participants have non-syndromic deafness, no participant became deaf due to systemic causes that also affect vision and no participant received a cochlear implant (CI). Information on the type of hearing loss (i.e. sensorineural, conductive or mixed) was not available. Five deaf participants never used hearing aids, 10 deaf participants had used hearing aids in the past and the remaining 11 deaf participants were currently using hearing aids. The deaf participants’ self-rated speech comprehension with a hearing aid varied from poor to very good (Supplementary Table 1). Nine participants reported benefits with hearing aids, especially benefiting communication, 12 participants reported limited benefits with hearing aids, i.e. benefits occurred only for sound detection and awareness, and 5 participants never used hearing aids. Their speech articulation ability was on the floor: they could at most speak simple words with poor intelligibility. All the deaf participants were proficient in Chinese Sign Language at the time of the experiment. The experimenter was a hearing signer who was proficient both in Chinese spoken and in sign language. The experimenter communicated with the deaf participants mainly through Chinese sign language. The normal-hearing participants had no known hearing deficits and reported normal comprehension of speech in everyday situations, and all of them are native Chinese speakers. Both groups of participants were right-handed as assessed by the 10-item version of the Edinburgh-Handedness-Inventory (EHI),^47^ with normal or corrected-to-normal vision and normal colour vision. None of them had a history of neurological or psychiatric disorders. All participants had given informed consent before the experiment following the Helsinki declaration and got paid for their participation afterwards. The Ethics Committee of the School of Psychology, South China Normal University, approved this study.

**Table 1:** Demographics of the two groups of participants participating in the present study

	Hearing (*n* = 24)	Deaf (*n* = 26)	Statistics	*P*-value
Gender, male/female	12/12	12/14	*χ*² = 0.074	0.786
Age, years (SD)	21.6 ± 1.7	21.5 ± 2.1	*t* = 0.084	0.933
Age of onset of hearing aid use, years (SD)		15 ± 4.9		
Degree of hearing loss (dB)				
*Left ear*		105 ± 13		
*Right ear*		107 ± 13		

### Stimuli and experimental set-up

The visual stimuli were projected via an LCD system onto a rear projection screen located behind the participants’ heads. Participants viewed the screen through an angled mirror on the MR head coil. The stimuli consisted of a fork (2.5° of visual angle in width) on the top of an orange plate (15° of visual angle in diameter) displayed on a grey background (Fig. 1B). The fork in each trial was either light or dark grey (red–green–blue value: 64–64–64 versus 192–192–192). The fork’s egocentric positions (concerning the participant’s mid-sagittal) and allocentric positions (concerning the plate’s mid-sagittal) were orthogonally varied (Fig. 1B). The visual angles of the egocentric and allocentric positions of the targets were chosen via a previous psychophysical test using another group of hearing participants to balance the task difficulty between the allocentric and egocentric judgements in the hearing controls. These chosen allocentric and egocentric positions were effective in balancing the task difficulty across the three experimental tasks in the normal-hearing controls (see the ‘Results’ section).

### Experimental tasks and designs

The experiment was scripted and run by Presentation software (Neurobehavioral Systems, RRID: SCR_002521, https://www.neurobs.com/). Participants were asked to perform three types of tasks on the same set of stimuli, i.e. allocentric, egocentric and non-spatial luminance discrimination tasks. In the allocentric judgement task (ALLO), participants judged whether the fork was on the left or the right side of the plate. In the egocentric judgement task (EGO), participants judged whether the fork was on the left or the right side of their mid-sagittal plane. Participants judged whether the fork was light grey or dark grey in the non-spatial luminance judgement task (as the high-level baseline, HLB). Two response pads, placed on the participants’ left and right sides, were used. For the spatial judgement tasks, participants pressed one button on the left response pad with their left thumb for the ‘left-side’ judgement and another button on the right response pad with their right thumb for the ‘right-side’ judgement. For the HLB task, participants pressed the left or right button with their left or right thumb corresponding to the light grey or the dark grey judgement. The mapping between the two kinds of luminance and the two response buttons was counterbalanced across participants. Therefore, the experimental design was a 2 (between-subject factor: deaf versus hearing) × 3 (within-subject factor, type of task: ALLO, EGO and HLB) two-factorial design.

The three types of tasks were blocked, and an event-related fMRI design was embedded with each block (Fig. 1C). Participants alternated among the three types of task blocks, and there was no rest block between the task blocks. There were 10 blocks in total for each type of experimental task. For each participant, a pseudo-random order of all the task blocks was given to ensure that the most extended time interval between any two blocks of the same type did not exceed 200 s, to meet the high-pass filter of 1/200 Hz for the preprocessing of the task-state fMRI data. Within each block, an instruction was first displayed for 3.3 s, informing the participants of the type of task to be performed in the upcoming block, and then 16 experimental trials and 6 null trials were presented, resulting in a block duration of 50.6 s. In each experimental trial, the target was presented for 250 ms, and such a short stimulus presentation time was chosen to minimize eye movements. In the null trials, only a blank default screen was presented. The inter-trial intervals (ITIs) were jittered from 1400 to 2400 ms with a step of 250 ms (i.e. 1650, 1900, 2150 and 2400 ms) with a mean ITI of 2000 ms. There were 660 trials in total, consisting of 480 experimental trials and 180 null trials. The whole task experiment lasted for 26.73 min.

We chose not to use a central ﬁxation because if a central ﬁxation cross was presented throughout the experiment, participants might use the position of the central ﬁxation, rather than the mid-sagittal plane of their own body, as an allocentric reference object to perform the egocentric task. Instead, we intended to force the participants to implement their mid-sagittal plane and use it during the egocentric task blocks. Nevertheless, participants were required to keep their gaze straightforward. The importance of not moving their eyes was repeatedly emphasized. Eye-tracking data from our previous study, in which the same experimental paradigm was adopted, suggested that central fixation can be maintained equally well in the allocentric and egocentric tasks.^9^ Before the formal experiment, all the participants completed a training session with ‘correct’ or ‘wrong’ feedbacks to familiarize themselves with the experimental tasks, and they were allowed to enter the formal experiment after they reached an accuracy rate above 90% for all the three tasks. On average, the training session lasted no more than 10 min for each participant, due to the three tasks’ simplicity.

### Data acquisition

The brain imaging data were acquired using a 3.0 T Siemens Trio Tim MRI scanner with a 32-channel head coil at the Institute of Psychophysics, Chinese Academy of Sciences, Beijing. Three scanning sequences were acquired: first, a resting-state fMRI session, then a task-state fMRI session and a structural T1-weighted MRI session. The resting-state and the task-state scanning adopted the same imaging parameters with T2*-weighted echo-planar imaging (EPI) sequence: repetition time (TR) = 2.2 s, echo time (TE) = 30 ms, flip angle (FA) = 90˚, matrix size = 64 × 64, voxel size = 3.44 × 3.44 × 3.0 mm^3^. During each TR, 36 transversal slices covering the whole brain with a 0.75 mm gap were acquired. The resting-state scan lasted for 7.33 min (one run of 200 continuous EPI volumes) and participants were instructed to close their eyes and not think of anything in particular. The task-state scanning lasted for 26.73 min (one run of 729 EPI volumes). The structural scans covering the entire brain were acquired for each participant using a T1-weighted 3D sagittal magnetization prepared rapid-acquisition gradient-echo pulse sequence with the following parameters: TR = 2530 ms, TE = 3.37 ms, inversion time = 1100 ms, FA = 7°, matrix size = 256 × 192, slice thickness = 1.33 mm, gap = 0, number of slices = 144. The T1 scan lasted for 8.09 min.

### Analysis of the behavioural data

For each experimental condition, missed trials, error trials with incorrect responses and outlier trials with reaction times (RTs) shorter or longer than ‘mean RT ± three times the standard deviation (SD)’ were excluded from further analysis. The proportions of the outlier trials were: 1.7% of the allocentric trials, 1.5% of the egocentric trials and 1.5% of the HLB trials in the deaf group; 1.7% of the allocentric trials, 1.7% of the egocentric trials and 1.6% of the HLB trials in the hearing group. Considering RTs are not distributed normally but show a positive skew,^48,49^ a generalized linear mixed model (GLMM) analysis was performed with gamma distribution (link = log) in R 4.1.2 (R core team 2021; lme4 package).^50^ The participant group (deaf versus hearing), the type of tasks (ALLO, EGO and HLB) and their interaction were modelled as fixed factors, the RT of each valid trial was the dependent variable and the random intercept effect structured by participants was also included. For the accuracy data, a GLMM with binomial distribution (link = logit) was used with type of response (correct or incorrect) specified as the dependent variable. The fixed and random factors were the same as the RT analysis. The main effects and the interaction were obtained from the fitted GLMM by using the ‘car’ package (type III),^51^ and the simple effect was obtained by using the ‘emmeans’ package^52^ with the Bonferroni correction for multiple comparisons, if there was a significant interaction.

Note, all the GLMM analyses were performed at the single-trial level. Only for demonstration purposes, the mean RTs and mean error rates were calculated and shown as a function of the two groups in the behavioural data figures. No further statistical analysis was performed on the averaged data.

### Analysis of the task-state fMRI data

#### Preprocessing

fMRI data of the task-state were processed with Statistical Parametric Mapping software SPM12 (Wellcome Department of Imaging Neuroscience, London, http://www.fil.ion.ucl.ac.uk) running on MATLAB R2014b (The MathWorks Inc., USA). The first five volumes were first discarded to allow for T1 equilibration effects. The remaining images were then spatially realigned to the new first volume to correct for inter-scan head movements. Then, images were normalized to the standard Montreal Neurological Institute (MNI) space and were resampled to 3 × 3 × 3 mm^3^. Finally, the normalized images were smoothed with a Gaussian kernel of 6 mm full-width half-maximum (6 mm FWHM) to accommodate inter-subject anatomical variability.

#### Statistical analysis

Data were high-pass-filtered at 1/200 Hz and modelled using the general linear model (GLM) as implemented in SPM12. At the first level, the GLM was used to construct a multiple regression design matrix. Three experimental conditions, i.e. ‘EGO’, ‘ALLO’ and ‘HLB’, were modelled in an event-related analysis. The three types of transient neural events were time-locked to the onset of each trial’s target by a standard HRF and its first-order time derivative (TD) with an event duration of 0 s. Besides, all the instructions, all the behaviourally missed trials, error trials and outlier trials were separately modelled as another regressor of no interest. The null trials were not modelled and treated as the implicit baseline in the GLM model. The six head movement parameters derived from the realignment procedure were included as confounds. Temporal autocorrelation was modelled using an AR (1) process. Parameter estimates were calculated for each voxel using weighted least-squares to provide maximum-likelihood estimators based on the data’s temporal autocorrelation. For each participant, simple main effects for each of the three experimental conditions were computed via appropriate ‘1 0’ baseline contrasts (i.e. experimental trials versus implicit baseline in the null trials) by putting ‘1’ on one experimental condition and ‘0’s on all the other regressors. At the group-level analysis, a 2 (between-subject factor: deaf versus hearing) × 3 (with-subject factor, type of task: ALLO versus EGO versus HLB) ANOVA was calculated by employing a random-effects model (full factorial design in SPM12). For conjunction analyses, the conjunction null hypothesis, instead of the global null hypothesis, was tested as implemented in SPM12.^53,54^ To localize the neural regions which were specifically involved in the egocentric or the allocentric task in the deaf group, when compared with the hearing group, exclusive masking procedures were adopted to avoid the potential contribution of the other task of no interest in the hearing controls. Specifically, the ‘Deaf (EGO > ALLO)’ contrast was exclusively masked by the ‘Hearing (EGO > ALLO)’ contrast, at a liberal threshold of *P* < 0.05, uncorrected at the voxel level. Via this exclusive masking procedure, any significant activations in the latter mask contrast, at a liberal threshold of *P* < 0.05, uncorrected at the voxel level, were excluded from further analysis in the former contrast. In this way, the neural regions, which showed significantly higher neural activity in the egocentric than allocentric task only in the deaf group, but a smaller or no effect in the hearing group, were localized. Similarly, the neural contrast ‘Deaf (ALLO > EGO)’ was exclusively masked by the neural contrast Hearing (ALLO > EGO), at a liberal threshold of *P* < 0.05, uncorrected at the voxel level. Areas of activation were identified as significant only if they passed a conservative threshold of *P* < 0.05, FWE correction for multiple comparisons at the cluster level with an underlying voxel level of *P* < 0.001, uncorrected.^55^

#### Psychophysiological interaction analysis

To further investigate how congenital deafness altered the within- and between-network dynamics in the two task-positive networks (i.e. DAN and FPN) and the task-negative network (i.e. the DMN), as a function of the egocentric versus allocentric task, we further performed the psychophysiological interaction (PPI) analysis to estimate the context-specific functional modulation of neural activity across the whole brain. The PPI analysis allows for detecting regionally specific responses in one brain area regarding the interaction between input from another brain region and a cognitive–sensory process.^56^ The bilateral inferior temporal gyrus (ITG) in the DAN, which was commonly involved in representing visuospatial positions concerning both reference frames (see the ‘Results’ section), the left posterior parietal cortex (PPC) in the FPN, which showed hyperactive neural activity (see the ‘Results’ section), and the medial prefrontal cortex (mPFC) in the DMN, which showed hyper-deactivated neural activity during the egocentric judgement in deaf participants (see the ‘Results’ section), were used as the seed regions (i.e. the physiological factor), respectively, and the ‘egocentric versus allocentric task’ was used as the psychological factor. Specifically, individual peak seed regions in the bilateral ITG were selected from the conjunction between the ‘EGO > HLB’ and the ‘ALLO > HLB’ contrast in each participant; individual seed regions in the left PPC were selected from the neural contrast ‘EGO > ALLO’ at the individual level; and individual seed regions in the mPFC were selected from the neural contrast ‘ALLO > EGO’ at the individual level. Each participant’s peak voxel was determined as the maximally activated voxel within a sphere of 16 mm radius around the coordinates of the group peak voxel from the second-level analysis. Consequently, the individual peak voxels were well located around these seed regions (left ITG MNI: *x* = −52 ± 5, *y* = −62 ± 8, *z* = −8 ± 6; right ITG MNI: *x* = 55 ± 5, *y* = −53 ± 7, *z* = −9 ± 6, left PPC MNI: *x* = −15 ± 5, *y* = −70 ± 5, *z* = 56 ± 5 and mPFC MNI: *x* = 8 ± 5, *y* = 55 ± 6, *z* = 9 ± 5).

After the individual seed voxels were selected, time series were extracted from a 4 mm radius around the individual peak voxels, as the physiological factors. PPI analysis at the individual level employed three regressors: (i) the physiological variable of interest (i.e. the time series extracted from the bilateral ITG, the left PPC and the mPFC); (ii) the psychological variable of interest (i.e. ‘EGO > ALLO’); and (iii) the cross-product of the previous two (i.e. the PPI term). An SPM was calculated to reveal the areas in which the neural activation was predicted by the PPI term, with the physiological and the psychological regressors treated as confound variables. The PPI analysis was carried out for each participant and then entered into a random-effects group analysis with the RT difference between the egocentric and allocentric tasks (‘EGO_RT > ALLO_RT’) as a covariate for each group. The behavioural covariate was included in the second-level group model to test whether and how the functional connectivity between the seed region and the other brain regions changed during the egocentric task due to individual differences in egocentric performance. The relative RT difference between the egocentric versus allocentric task (‘EGO_RT > ALLO_RT’), rather than the absolute egocentric RTs, was included as the covariate because we aimed to test the specific effect of the egocentric performance *per se*, rather than the effect of general response speed, which is characterized by the absolute RTs in all three tasks. Therefore, by contrasting the absolute RTs in the egocentric versus allocentric task in each participant, the effect of general response speed was cancelled out, and only the clean and specific effect of the egocentric performance was derived. The PPI activations were reported as significant at a conservative threshold of *P* < 0.05, FWE correction for multiple comparisons at the cluster level with an underlying voxel level of *P* < 0.001, uncorrected.^55^ Only for the seed region in the left PPC, significant PPI activations were reported at a less conservative threshold of *P* < 0.05, FWE correction at the cluster level with an underlying voxel level of *P* < 0.005, uncorrected, mostly for demonstration purposes. Although the left PPC’s significance threshold is less conservative than the other seed regions in the present experiment, it is still strict enough to control for false-positive errors.^56^ All the key activations based on the left PPC seed region survived the conservative threshold at *P* < 0.05, FWE correction for multiple comparisons at the cluster level with an underlying voxel level of *P* < 0.001, uncorrected, as well for both groups.

### Analysis of the resting-state fMRI data

#### Preprocessing

Resting-state fMRI data were preprocessed using the DPARSF module of the DPABI pipeline (a toolbox for Data Processing and Analysis of Brain Imaging; http://www.rfmri.org). The first five volumes were discarded. Then slice timing was performed, and the corrected time series were realigned to the new first volume for head motion correction. The fMRI images were normalized into MNI space using new segmentation and DARTEL and then resampled to 3 mm isotropic voxels. Nuisance covariates, including Friston-24 parameters of head motion, white matter and cerebrospinal fluid mean signals, were regressed out. The preprocessed time series were then filtered with a temporal band-pass of 0.01–0.1 Hz. The global signal was not regressed out since the whole-brain signal regression could exaggerate negative correlations.^57–59^

#### Network construction

To constrict the network node definition within the brain regions that were involved in the present three experimental tasks, we created a task mask by running an *F*-test on the ‘allocentric versus egocentric versus HLB’ contrast (collapsed over the two groups), at the statistical threshold of *P* < 0.01, FWE correction at the cluster level with an underlying voxel-level threshold at *P* < 0.001, uncorrected (see the ‘Results’ section). In this way, any two-tail differential activation between any two of the three experimental tasks was included in the task mask. Subsequently, the pre-defined task mask was applied to extract the resting-state fMRI data on the individual level. For each participant, time courses of the resting-state data were first extracted from each voxel in the task mask, and the voxel-wise Pearson correlation matrix was computed. The resultant correlation matrices were then thresholded to generate binary brain graphs, from 1 to 5% connection density with a step of 1%. The lowest threshold (1%) was adopted to make sure that the resulting graphs are not severely fragmented (the largest component size > 90%), while the highest threshold (5%) was chosen to remove weak correlations so that only the correlations with *P*-values passed a statistical threshold (*P* < 0.05) were retained. The *P*-values were corrected for multiple comparisons with the false discovery rate (FDR) procedure at a *q*-value threshold of 0.05. Since negative correlations accounted for only a tiny portion of the overall voxel-wise correlation matrices after the thresholding procedure, irrespective of whether the global signal was removed or not, we only focused on the positive connections in the following analysis by setting the negative correlations to zero.

#### Modularity analyses

The thresholded brain graphs were then subjected to a graph-based modularity analysis to identify brain modules (i.e. brain networks) and estimate module-based graph properties via the GRETNA toolbox.^60^

To identify modules (i.e. groups of nodes that are highly connected to each other and less connected to the other nodes), we adopted the following module identification algorithm in which the modularity, *Q*, was defined as:

$$Q=\overset{M}{\underset{s=\;1}{\sum }}\;[l_{s}/L-(d_{s}/2L{)}^{2}],$$

where *M* is the number of modules, *l_s_* the number of within-module edges in the module *s*, *L* the total number of edges in the network, *d_s_* the sum of the degrees at each node in the module *s* and the degree of a node is the number of linked edges within the given node.^61,62^

The modularity analysis was first performed on each individual brain graph in both groups. The module number and membership varied among participants within the same group and across the two groups. Therefore, to first test whether the two groups showed reliable and similar module structure, we computed the similarity between the hearing and the deaf group using the adjusted mutual information (AMI),^63^ a measure of the similarity between two partitions that ranges from 0 for unrelated partitions to 1 for identical partitions. A group brain graph was first calculated for each group by averaging all the individual correlation matrices within the group and then thresholding from 1 to 5%. Subsequently, the AMI values were computed between the group brain graphs in the two groups across different density thresholds. Since the modular partition was very similar between the two groups according to the AMI analysis (see the ‘Results’ section), we performed the modularity analysis on the group-level brain graph (collapsed over the two groups) to determine a general modular structure shared by all the participants, at each of the pre-selected network densities from 1 to 5%. To further compute the module-based graph properties at both the module and the nodal level, the two task-positive networks in the DAN and the FPN and the task-negative network in the DMN, which were of particular interest in the present study, were selected by visual inspection from the group-level modularity analysis (two groups combined), at the network density of 3%.

At the module level, the intra-module connectivity was calculated as the sum of positive connections within a module, while the inter-module connectivity was calculated as the summed number of positive connections between any pair of two modules (‘DAN–FPN’, ‘DMN–DAN’ and ‘DMN–FPN’).

At the nodal level, the within-module degree (WD) *z*-score and the participation coefficient (PC) between any pair of two modules between the FPN, the DAN and the DMN were computed.^64,65^ The WD measures the normalized degree of connections of a node within its corresponding module:

$$z_{i}=\frac{k_{i}\;-\;\bar{k_{s}}}{\sigma_{s}},$$

where *k_i_* is the number of intra-module connections of a node *i* within module *s*, *k_s_* the average number of intra-module connections of all the nodes in module *s* and *σ_s_* the standard deviation of the number of intra-module connection at all the nodes in module *s*. Thus, z*_i_* is higher for a node with a larger number of intra-module connections than the other nodes in the same module.

Since we were particularly interested in the changes in the PC between the task-positive networks (‘FPN–DAN’) and the task-negative DMN (‘DAN–DMN’ and ‘FPN–DMN’), the PC was calculated only between the three pairs of modules. Specifically, for each pair of two modules, the PC for node *i* is deﬁned as follows:

$$PC_{i}=1-\overset{N_{M}}{\underset{s=\;1}{\sum }}\;{\left(\frac{k_{is}}{k_{i}}\right)}^{2},$$

where *N*_M_ is the number of modules (i.e. ‘2’ for the present analysis), *k_is_* the number of connections between the node *i* and the module *s* and *k_i_* the total number of connections of node *i* in the whole network (including two modules in the present analysis). The PC of node *i* will be close to ‘1’ if its connections are distributed among different modules and ‘0’ if connected exclusively within its own module.

#### Statistical analysis

For the module-wise measures (i.e. the number of intra-/inter-module connections), planned two-sample *t*-tests were used to test the between-group difference. For the nodal-wise (i.e. voxel-wise) analysis, individual (WD or PC) images from the two groups were submitted to a second-level two-sample *t*-test group analysis as implemented in SPM12. The egocentric performance (‘EGO_RT > ALLO_RT’) of each participant was also included as a covariate in the second-level model. Areas of activation were defined as significant if they passed a statistical threshold of *P* < 0.05, FWE correction for multiple comparisons at the cluster level with an underlying voxel level of *P* < 0.05, uncorrected.

#### Validation analysis

To evaluate the reliability and reproducibility of our results, we examined the influences of different preprocessing strategies (with versus without removal of global signal) and network density thresholds (3 versus 4%).

### Data availability

The data that support the findings of this study are available from the corresponding author upon reasonable request.

## Results

### Impaired body-centred sensorimotor transformation in the deaf group

Consistent with our hypothetical model (Fig. 1A), the interaction between the participant group and the type of tasks was significant (*χ*^2^ = 164.06, *P* < 0.001). Specifically, egocentric processing was impaired in the deaf group: egocentric performance was significantly slower in the deaf [emm = 6.47, CI = (6.41; 6.54)] than the hearing control group [emm = 6.37, CI = (6.31; 6.44); *z* = 2.23, *P* < 0.05]. No significant between-group difference was found neither in the allocentric task nor in the non-spatial HLB task, both *P*s > 0.05. For the accuracy data, the interaction between the group and the type of tasks was significant as well (*χ*^2^ = 13.20, *P* < 0.01). Further analysis on the simple effects showed that there were more errors in the egocentric task [emm = 2.48, CI = (2.27; 2.70)] than in both the allocentric task [emm = 2.95, CI = (2.72; 3.18); *z* = −5.26, *P* < 0.001] and the non-spatial HLB task [emm = 2.85, CI = (2.63; 3.08); *z* = −4.28, *P* < 0.001] in the deaf group, but the tasks did not significantly differ with respect to accuracy in the hearing group (all *P*s > 0.05). Therefore, the results of the accuracy data were consistent with the RT data, showing that egocentric processing was impaired in the deaf group. For demonstration purposes, the mean RT and the mean error rate across the participants are displayed in Fig. 1D and E, respectively. See the Supplemental Results for more detailed analyses of the behavioural data.

**Figure 2:**
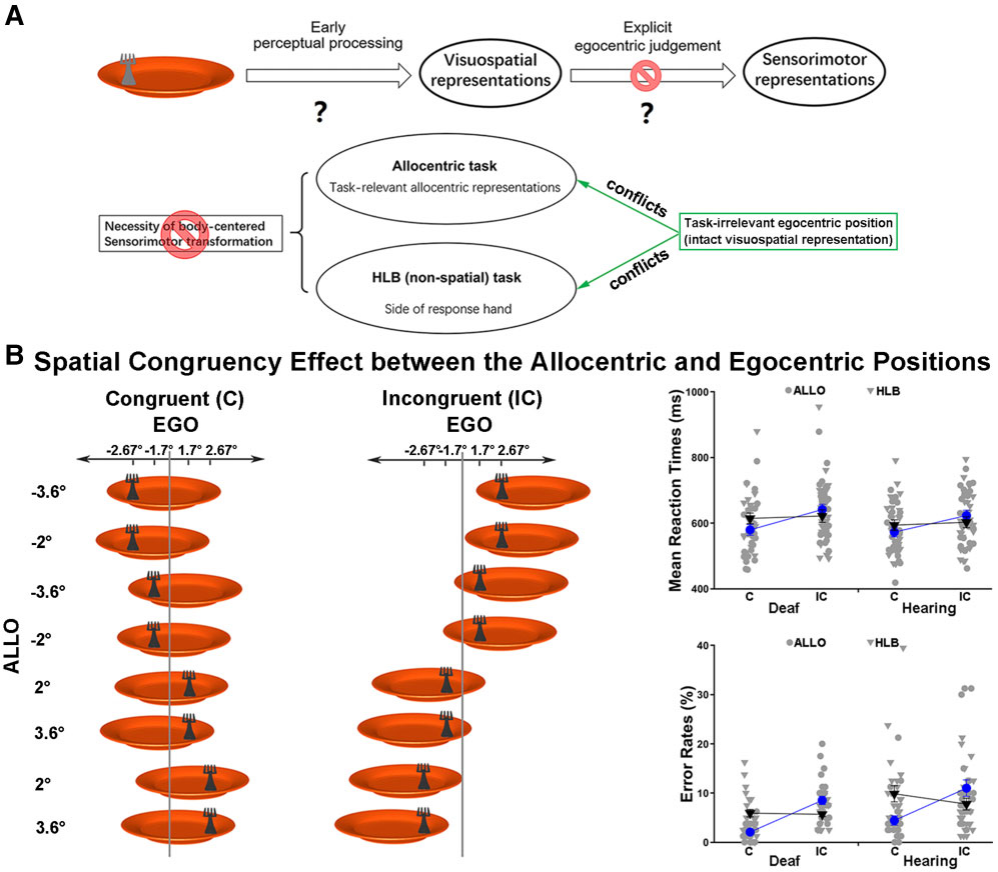
**Further analysis of the behavioural data on whether the fidelity of visuospatial representation of the egocentric positions or the explicit body-centred sensorimotor transformation is impaired by congenital deafness.** (**A**) Hypothesis and predictions. (**B**) Spatial congruency effect between the allocentric and egocentric positions. *Left panel*: the fork’s allocentric and egocentric position could be either congruent (both left or both right) or incongruent (one left and one right). All the possible stimulus combinations in the congruent versus incongruent condition were shown for the dark grey fork as an example. For demonstration purposes, the mean RTs (*top*) (congruent versus incongruent: ALLO, *z* < −11.5, *P* < 0.001; HLB, *χ*^2^ < 2.5, *p* > 0.05, GLMM) and error rates (*bottom*) (congruent versus incongruent: ALLO, *z* > 7.5, *P* < 0.001; HLB, *χ*^2^ < 3.5, *p* > 0.05, GLMM) are shown as a function of the congruent versus incongruent condition in both groups’ allocentric and HLB tasks (*right panel*).

In the egocentric task, the egocentric position of the fork has to be coded first as visuospatial representations during early perceptual processing and is then transformed into body-centred sensorimotor representations to allow for adequate sensory-guided actions (Fig. 2A, the top panel). Both processes may have contributed to the slowed performance of the deaf participants in the egocentric task.

**Figure 3:**
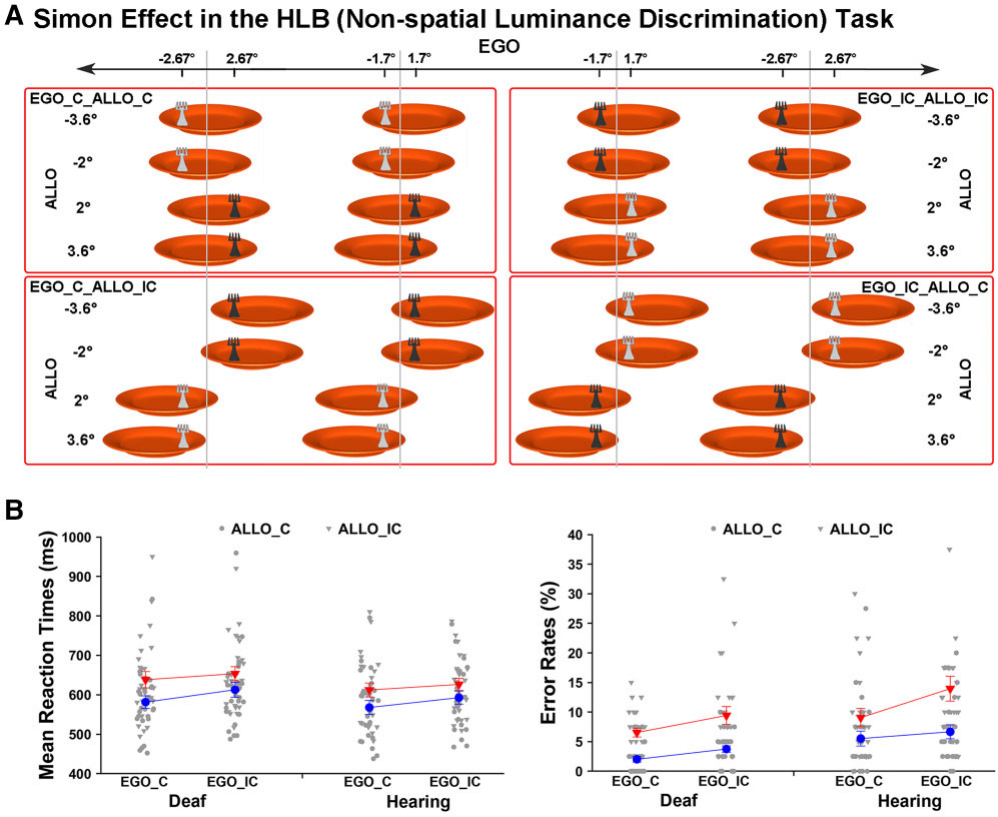
**Simon effect in the HLB (non-spatial luminance discrimination) task.** (**A**) In the non-spatial HLB task, the allocentric and egocentric positions were both task-irrelevant and could be either congruent (ALLO_C, EGO_C) or incongruent (ALLO_IC, EGO_IC) with the response hand’s side, i.e. the Simon effect. The full stimulus set is shown under the condition when the left hand is assigned to the light grey response and the right hand to the dark grey response. (**B**) For demonstration purposes, the mean RTs (*left*) (congruent versus incongruent: ALLO, *χ*^2^ = 66.04, *P* < 0.001; EGO, *χ*^2^ = 23.75, *P* < 0.001, GLMM) and error rates (*right*) (congruent versus incongruent: ALLO, *χ*^2^ = 9.15, *P* < 0.01; EGO, *χ*^2^ < 1.5, *p* > 0.05, GLMM) are shown as a function of the Simon congruency effect based on the allocentric and egocentric positions in both groups. The error bars indicate standard errors (SEs).

The allocentric task and the HLB (non-spatial luminance discrimination) tasks helped to further disentangle the effects of these two processes, since these tasks did not demand body-centred sensorimotor transformations (Fig. 2A). If congenital deafness only causes deficits in the explicit body-centred sensorimotor transformation process, then the task-irrelevant (but intact) visuospatial representations of the egocentric positions should cause comparable spatial conflicts with the task-relevant allocentric positions in the allocentric task (Fig. 2B, the left panel) or with the side of the response hand in the HLB task (Fig. 3A) in both groups of participants (Fig. 2A, the bottom panel). Consistent with our predictions, the three-way interaction was significant (*χ*^2^ = 13.86, *P* < 0.001). Further analyses showed that in the allocentric task, when the necessity of body-centred sensorimotor transformation was abolished, the task-irrelevant egocentric positions caused significant spatial congruency effects on the task-relevant allocentric positions in both groups (all *P*s < 0.001). Additionally, the spatial congruent effect was significantly larger in the allocentric task than the egocentric task in the deaf group [*t*_(25)_ = 3.11, *P* < 0.01], but there was no significant difference in the hearing group (*P* > 0.05). Therefore, the visuospatial representations of the egocentric positions were well maintained in the deaf brain, when body-centred sensorimotor transformations were not required as in the allocentric task. However, the spatial congruency effect between the allocentric and egocentric positions was not evident in the HLB task, all *P*s > 0.05. Therefore, the spatial congruency effect between the two spatial reference frames does not occur when neither frame of reference was task-relevant in the non-spatial HLB task and thus is not simply induced by the slight difference in the bottom-up stimulus input (Fig. 2B, the left panel). The accuracy data showed the same pattern as the RTs. For demonstration purposes, the mean RT and the mean error rate across the participants were displayed as a function of the allocentric and the non-spatial HLB task (Fig. 2B, the right panel). Similarly, in the non-spatial HLB task, when the explicit spatial task demands (both egocentric and allocentric) were completely abolished, the task-irrelevant egocentric positions of the target caused significant and comparable conflicts on the side of the response hand, i.e. the classical Simon effect,^66,67^ in both the deaf and the hearing groups (*P* > 0.05). The accuracy data replicated the RT results in the HLB task. For demonstration purposes, the mean RT and the mean error rate across participants were shown as a function of the Simon congruency effect based on the allocentric and egocentric positions (Fig. 3B). See the Supplementary Results and Fig. 1 for all the detailed analyses of the behavioural data.

### Hyper-active left PPC in the FPN and deactivated mPFC in the DMN during egocentric processing in the deaf brain

**Figure 4:**
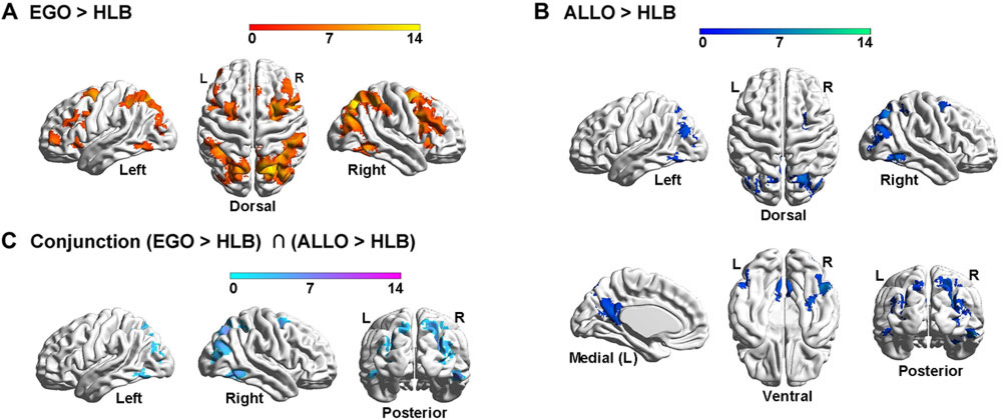
**Neural correlates underlying the allocentric and egocentric reference frame.** (**A**) Compared with the HLB task, the egocentric task activated an extensive bilateral occipital–parietal–frontal brain network. (**B**) Compared with the HLB task, the allocentric task activated both the bilateral ITG and the right hippocampus and the bilateral posterior dorsal occipital–parietal–frontal regions. (**C**) The conjunction analysis revealed the joint involvement of the classical DAN in both spatial tasks.

**Figure 5:**
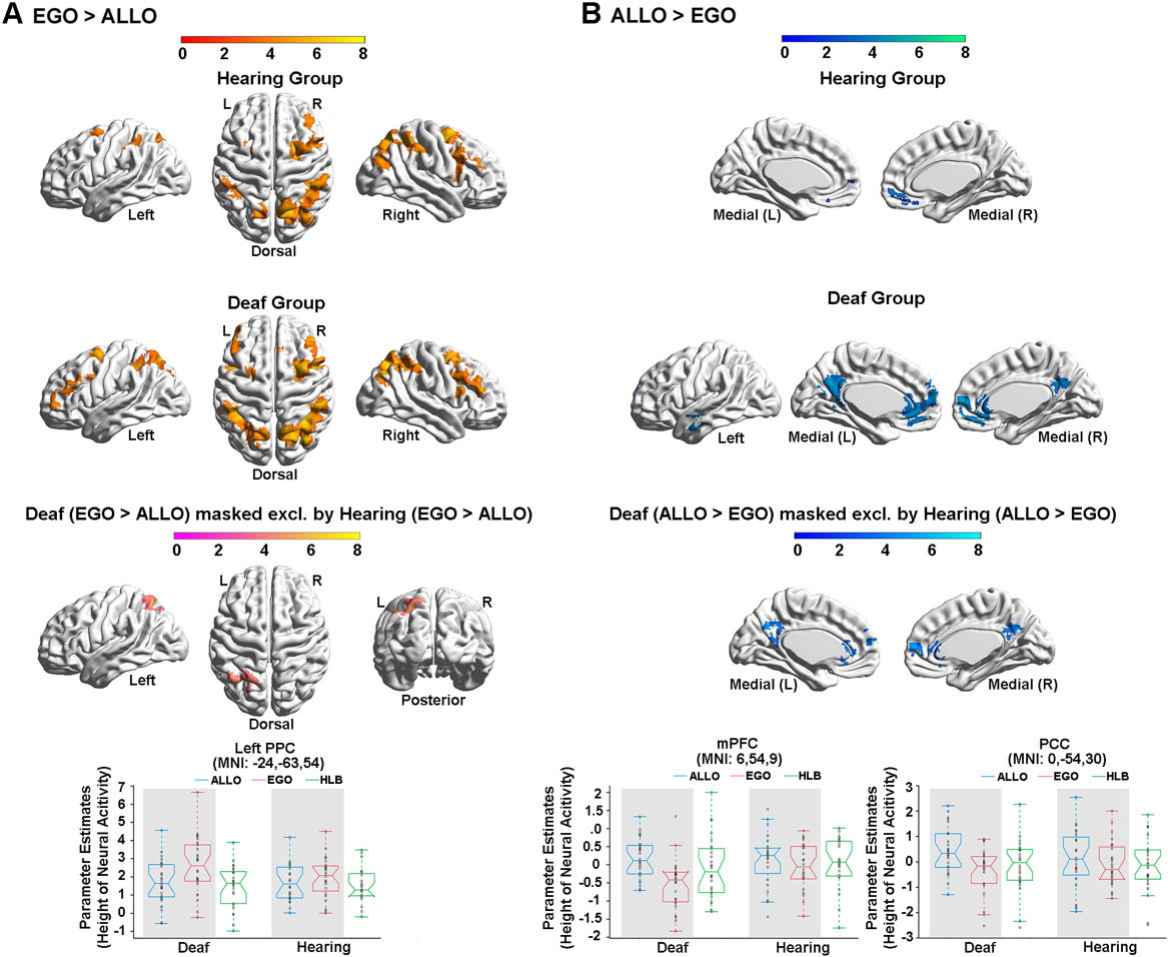
**Neural correlates underlying the allocentric and egocentric reference frame in the deaf and hearing groups.** (**A**) The neural contrast ‘EGO > ALLO’ was calculated in the hearing (*top*) and deaf (*middle*) group, respectively. *Bottom panel*: the left PPC in the FPN showed significantly higher neural activity in the egocentric than the allocentric task, specifically in the deaf group. (**B**) The neural contrast ‘ALLO > EGO’ was calculated in the hearing (*top*) and deaf (*middle*) group, respectively. *Bottom panel*: the mPFC in the DMN was significantly deactivated in the egocentric task, specifically in the deaf group (*left*). The PCC in the DMN showed significantly activated in the allocentric task, specifically in the deaf group (*right*). The mean parameter estimates in the three tasks were extracted from the left PPC, the mPFC and the PCC for both groups, no further statistical tests were performed to avoid double-dipping. The shaded conditions are the conditions involved in the neural contrast.

At the neural level, compared with the HLB task, the egocentric judgement task (Fig. 4A, Supplementary Fig. 2A and Table 2A) and the allocentric judgement task (Fig. 4B, Supplementary Fig. 2B and Table 2B) conjointly activated the classical DAN in the bilateral ITG and the middle-superior occipital gyrus extending to the posterior superior parietal cortex (the two groups combined) (Fig. 4C and Supplementary Table 2C). For both groups, neural activity in this commonly activated DAN was higher in the two spatial tasks than in the non-spatial HLB task (Supplementary Fig. 2C). Compared with the allocentric task, the egocentric task specifically activated the classical dorsolateral FPN (the two groups combined) (Supplementary Fig. 2D and Table 2D). Moreover, the deaf group showed more bilateral FPN activations than the right-lateralized FPN activations in the hearing group (Fig. 5A, the top and middle panels; and Supplementary Table 3C). Especially, the left PPC in the FPN was significantly more involved in the egocentric task in deaf than hearing participants (Fig. 5A, the bottom panel; and Supplementary Table 4A). Compared with the egocentric task, the allocentric task specifically activated the DMN, including the mPFC, posterior cingulate cortex (PCC), left angular gyrus (AG) and left middle temporal gyrus (MTG) close to the temporal pole (the two groups combined) (Supplementary Fig. 2E and Table 2E). Moreover, the DMN activations were more extensive in deaf than hearing participants (Fig. 5B, the top and middle panels; and Supplementary Table 3D). The mPFC region in the DMN was specifically more deactivated during the egocentric task in the deaf than hearing group (Fig. 5B, the box figure in the bottom left panel), and the PCC region in the DMN was specifically more positively activated during the allocentric task in the deaf than hearing participants (Fig. 5B, the box figure in the bottom right panel).

Therefore, two critical task-positive neural networks were involved in the human spatial reference frame system: (i) the DAN was commonly involved in representing the visuospatial representations of the allocentric and egocentric positions, compared with the HLB (Fig. 4C; Supplementary Table 2C); and (ii) the FPN was explicitly involved in the egocentric task, compared with the allocentric task (Fig. 5A and Supplementary Fig. 2D). In contrast, the DMN was identified as the task-negative network by showing either deactivated or close-to-zero neural activity during the egocentric task (Fig. 5B and Supplementary Fig. 2E).

### Altered neural network dynamics within and between the task-positive networks and the DMN during egocentric processing in the deaf brain

**Figure 6:**
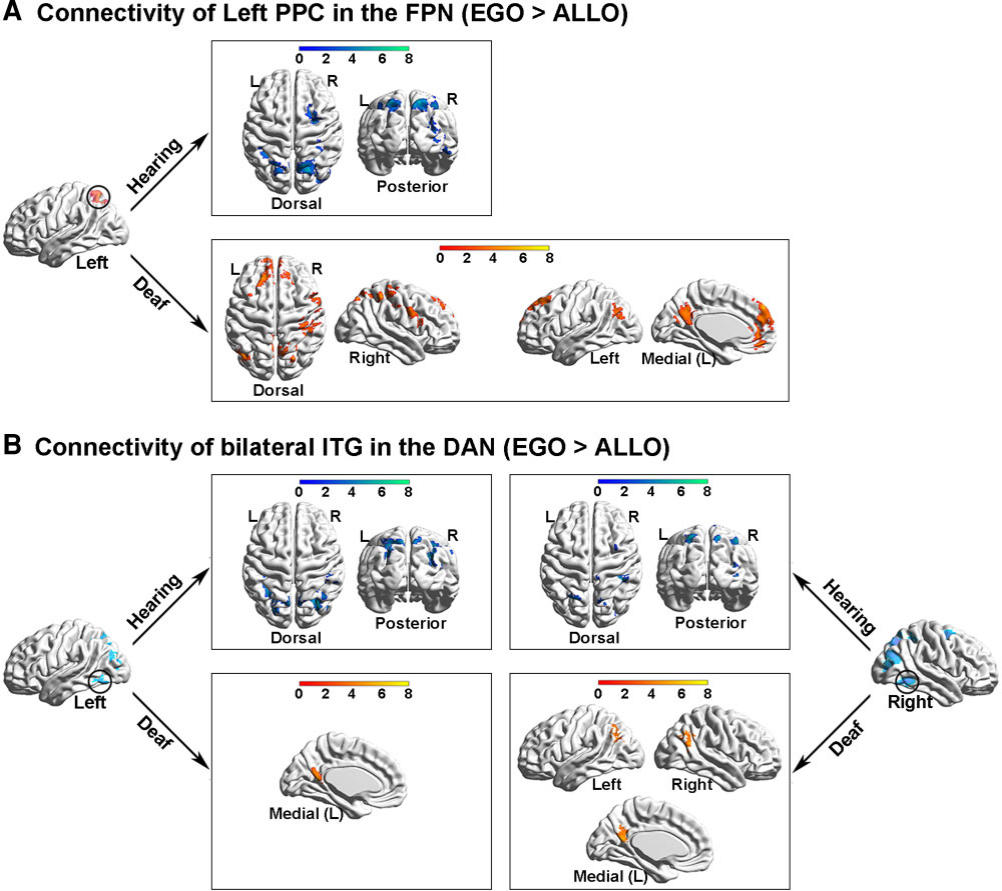
**PPI analysis on seed regions in the task-positive neural networks in the FPN and the DAN.** (**A**) PPI results with the left PPC in the FPN as the seed region. *Top panel*: in the hearing group, the left PPC showed significantly increased functional connectivity in the other task-critical occipital–parietal–frontal regions, specifically during the egocentric task, compared with the allocentric task. *Bottom panel*: in the deaf group, the left PPC showed significantly increased connectivity not only with the task-critical frontoparietal regions but also with the DMN subregions. (**B**) PPI results with the bilateral ITG in the DAN as the seed regions. *Top panel*: in the hearing group, both seed regions in the bilateral ITG showed significantly increased connectivity with the task-critical frontoparietal regions, specifically during the egocentric task, compared with the allocentric task. *Bottom panel*: in the deaf group, the bilateral ITG showed significantly increased connectivity with the DMN subregions.

**Figure 7:**
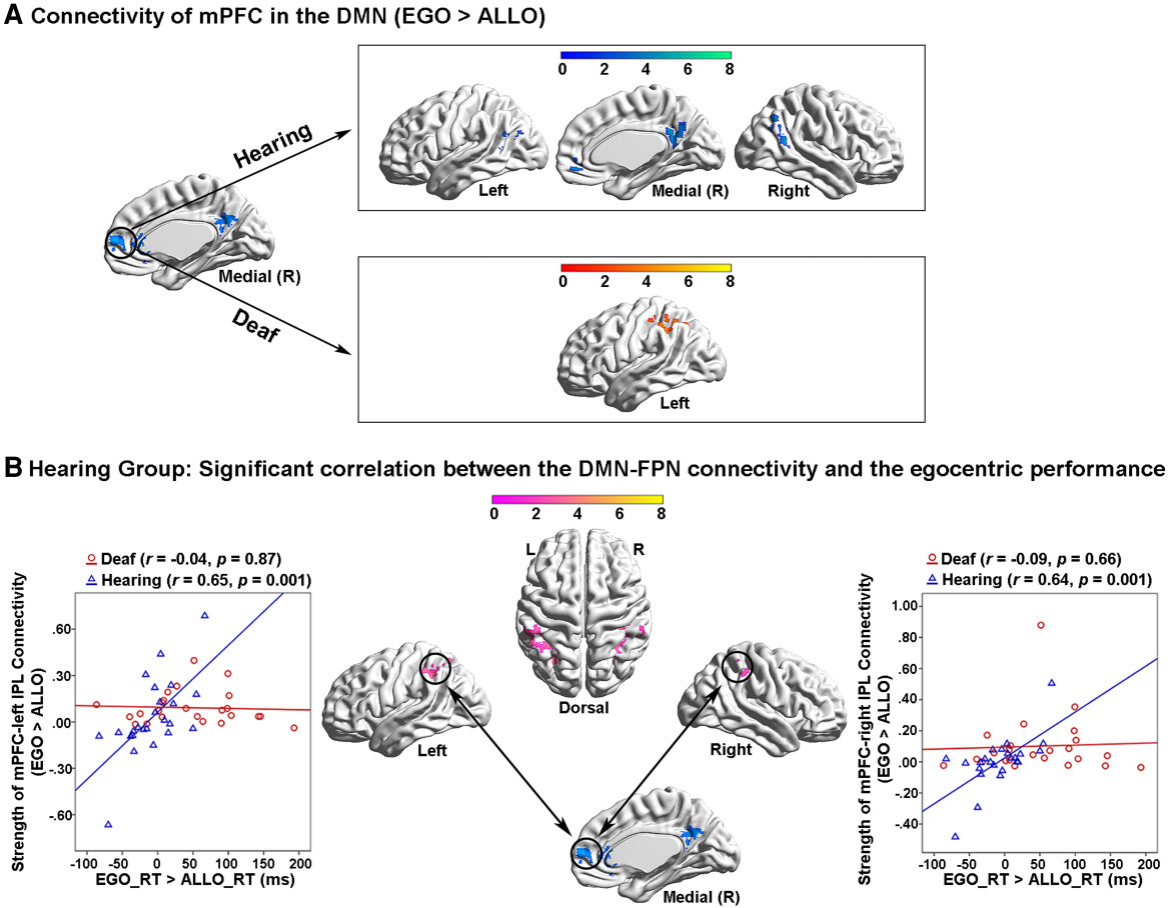
**PPI analysis with the mPFC in the task-negative DMN as the seed region.** (**A**) *Top panel*: in the hearing group, mPFC in the DMN showed significantly increased functional connectivity with the other subregions in the DMN, specifically during the egocentric task, compared with the allocentric task. *Bottom panel*: in the deaf group, mPFC in the DMN showed significantly increased functional connectivity with the left IPL in the FPN. (**B**) In the hearing group, the inter-network connectivity between the mPFC in the DMN and the bilateral IPL in the FPN was significantly positively correlated with the egocentric performance (EGO_RT > ALLO_RT). The stronger the inter-network connectivity between the DMN and the FPN during the egocentric task, the slower the egocentric judgements. This correlation was not significant in the deaf group.

In the hearing group, both the left PPC in the FPN and the bilateral ITG in the DAN showed significantly higher functional connectivity with the other task-positive occipital–parietal–frontal regions (Fig. 6A and B, the top panels; Supplementary Table 5A and B) while the mPFC in the DMN showed significantly higher functional connectivity with the other DMN subregions in the PCC, the bilateral AG and the orbital prefrontal cortex (Fig. 7A, the top panel; Supplementary Table 5C), in the egocentric than the allocentric task (i.e. the psychological factor ‘EGO > ALLO’). Moreover, in the hearing group, the covariate effect of the individual difference in egocentric performance (‘EGO_RT > ALLO_RT’) significantly involved the bilateral inferior parietal lobe (IPL) in the FPN (Fig. 7B; Supplementary Table 5D), indicating that the stronger the functional connectivity between the mPFC in the DMN and the bilateral IPL in the FPN during the egocentric task, the slower the egocentric performance. This correlation was not significant in the deaf group.

For the deaf group, the left PPC in the FPN showed significantly higher functional connectivity with not only the task-positive regions in the right ITG and the bilateral superior parietal lobe and IPL (Fig. 6A, the bottom left panel), but also with the task-negative DMN subregions in the left AG, the PCC and the mPFC (Fig. 6A, the bottom right panel; Supplementary Table 5A), in the egocentric than allocentric task (i.e. the psychological factor ‘EGO > ALLO’). The bilateral ITG in the DAN showed significantly higher functional connectivity with the subregions of the DMN as well, in the egocentric than allocentric task (Fig. 6B, the bottom panels; Supplementary Table 5B). Moreover, the mPFC in the DMN showed significantly higher functional connectivity with the left IPL in the FPN, in the egocentric than allocentric task (Fig. 7A, the bottom panel; Supplementary Table 5C).

Taken together, during egocentric processing, the coherent interaction within and between the task-positive neural networks (i.e. DAN and FPN) was intact in the deaf brain. However, compared with the hearing controls, the deaf participants showed (i) increased inter-network connectivity between the task-positive (DAN and FPN) and the task-negative (DMN) networks and (ii) decreased intra-network connectivity within the task-negative DMN.

### Similarly altered neural network dynamics during resting state in the deaf brain

**Figure 8:**
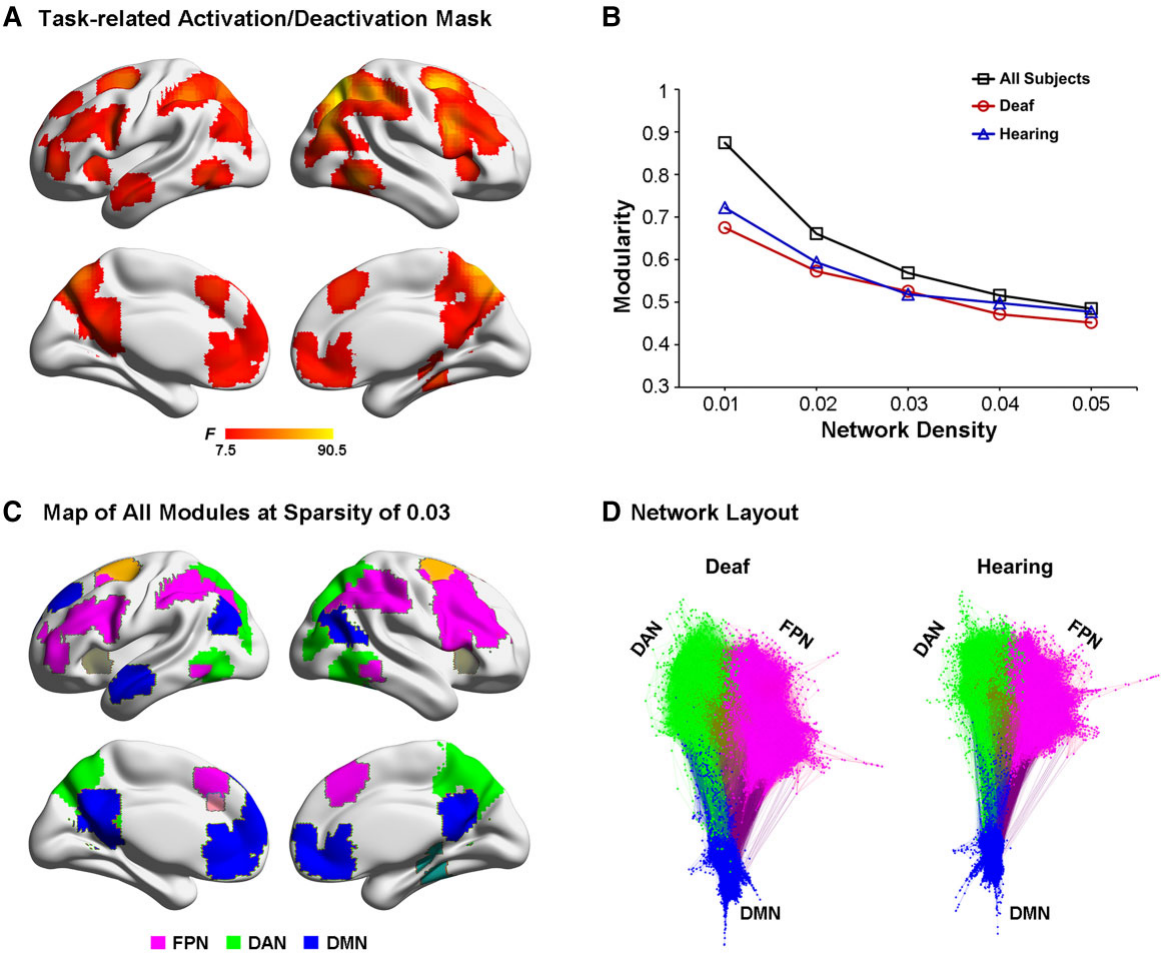
**Module identification of the resting-state data.** (**A**) Task-related activation or deactivation mask was derived via an *F*-test on two-tail comparisons between any two of the present three tasks, i.e. (‘ALLO versus HLB’) or (‘EGO versus HLB’) or (‘ALLO versus EGO’). (**B**) Mean modularity is shown as a function of the network sparsity from 1 to 5%, either in all participants, i.e. collapsed over all deaf and normal-hearing participants, or in the deaf and hearing group only. (**C**) Module partitions of the whole group brain graph (two groups combined), thresholded at the network density of 3%. The DMN (blue) and the two task-critical networks in the DAN (green) and the FPN (magenta) are clearly segregated. (**D**) A visual representation of the modularity difference between the deaf (*left*) and the hearing (*right*) group. This network layout is generated by a force-field algorithm. Within one module, smaller clusters indicate stronger intra-module connections, and vice versa. Between two modules, shorter distances indicate stronger inter-module connections, and vice versa. Most notably, the DMN (blue) is less segregated from the FPN (magenta) and the DAN (green) in the deaf than hearing group.

To further investigate whether the deaf brain maintains its characteristic network dynamics even during resting-state, i.e. in terms of the intrinsic neural activity, we run the following graph theory analysis on the resting-state fMRI data in the two groups, focusing on the intra- and inter-network connectivity between the task-positive networks in the DAN and the FPN, and the task-negative DMN (Fig. 8A).

In practice, a network’s modularity with a strong modular structure typically ranges from 0.3 to 0.7.^68^ Both the hearing and the deaf groups, respectively, and all the participants collapsed over the two groups showed high modularity *Q*-values across the density range (Fig. 8B). As the network density decreased, modularity *Q* increased monotonically. Moreover, for each density threshold, the AMI of module partitions in the two groups exhibited high and comparable values (ranging from 0.6 to 0.72), indicating the brain network’s module structure was relatively stable and similar between the two groups. Given the consistent module assignments between the hearing and the deaf group, we adopted the common module partitions based on the group brain graph collapsed over the two groups, thresholded at the network density of 3% (Fig. 8C). Nine modules were identified, including the FPN, the DAN and the DMN modules, which were selected for further analysis. The three selected modules’ network layout is depicted for the two groups, respectively, in Fig. 8D. It is most notable that the DMN was less segregated from the two task-critical networks in the FPN and the DAN in the deaf than the hearing group.

**Figure 9:**
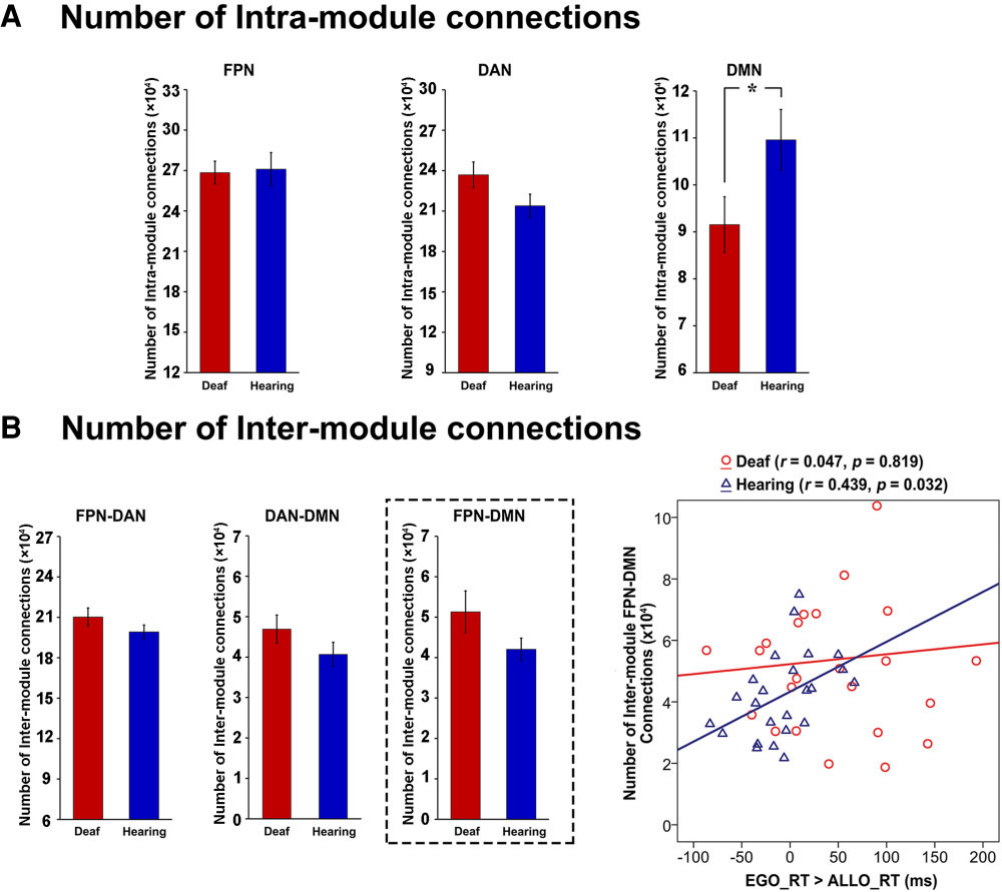
**Module-wise results of intrinsic neural activity during the resting state.** (**A**) The number of intra-module connections within the FPN [*t*_(48)_ = −0.17, *P* = 0.865], the DAN [*t*_(48)_ = 1.74, *P* = 0.088] and the DMN [*t*_(48)_ = −2.03, *P* = 0.048] is shown as a function of the two groups. (**B**) The number of inter-module connections between the FPN–DAN [*t*_(48)_ = 1.27, *P* = 0.211], the DAN–DMN [*t*_(48)_ = 1.33, *P* = 0.191] and the FPN–DMN (*t*_(48)_ = 1.51, *P* = 0.138) is shown as a function of the two groups. The between-group differences of the intra- and inter-module connections were test by planned two-sample *t*-tests. *The right-most panel*: the number of FPN-DMN inter-module connections significantly correlated with the egocentric performance in the hearing group. *P < 0.05.

At the module level, we tested whether the deaf and the hearing group differed in terms of the number of within-module (within-FPN, within-DAN and within-DMN) and between-module (FPN–DAN, DMN–DAN and DMN–FPN) connections. The results showed that the number of intra-module connections within the DMN was significantly lower in deaf than hearing participants, *t*_(48)_ = 2.03, *P* < 0.05 (Fig. 9A). None of the other between-group comparisons reached statistical significance (Fig. 9A and B). Interestingly, similar to the correlation results during the egocentric task in the hearing group (Fig. 7B), the number of inter-module connections between the FPN and the DMN during the resting state significantly correlated with the egocentric performance in the hearing group as well, *r* = 0.439, *P* < 0.05 (Fig. 9B, the right-most panel). The larger the number of the inter-module FPN-DMN connections at rest in an individual brain, the slower her/his egocentric (relative to allocentric) performance.

At the nodal level, we tested the between-group difference of each node’s (i.e. voxel) topological role within and between the FPN, the DAN and the DMN modules, and how the corresponding neural measures were correlated with egocentric performance. Two standard network metrics, within-module degree (WD) and PC, were used to depict the localization and diversity of connections linked to each node (see the ‘Materials and methods’ section). The WD measures the normalized degree of connections of a node within its corresponding module. No significant between-group difference was revealed in the WD metrics.

**Figure 10:**
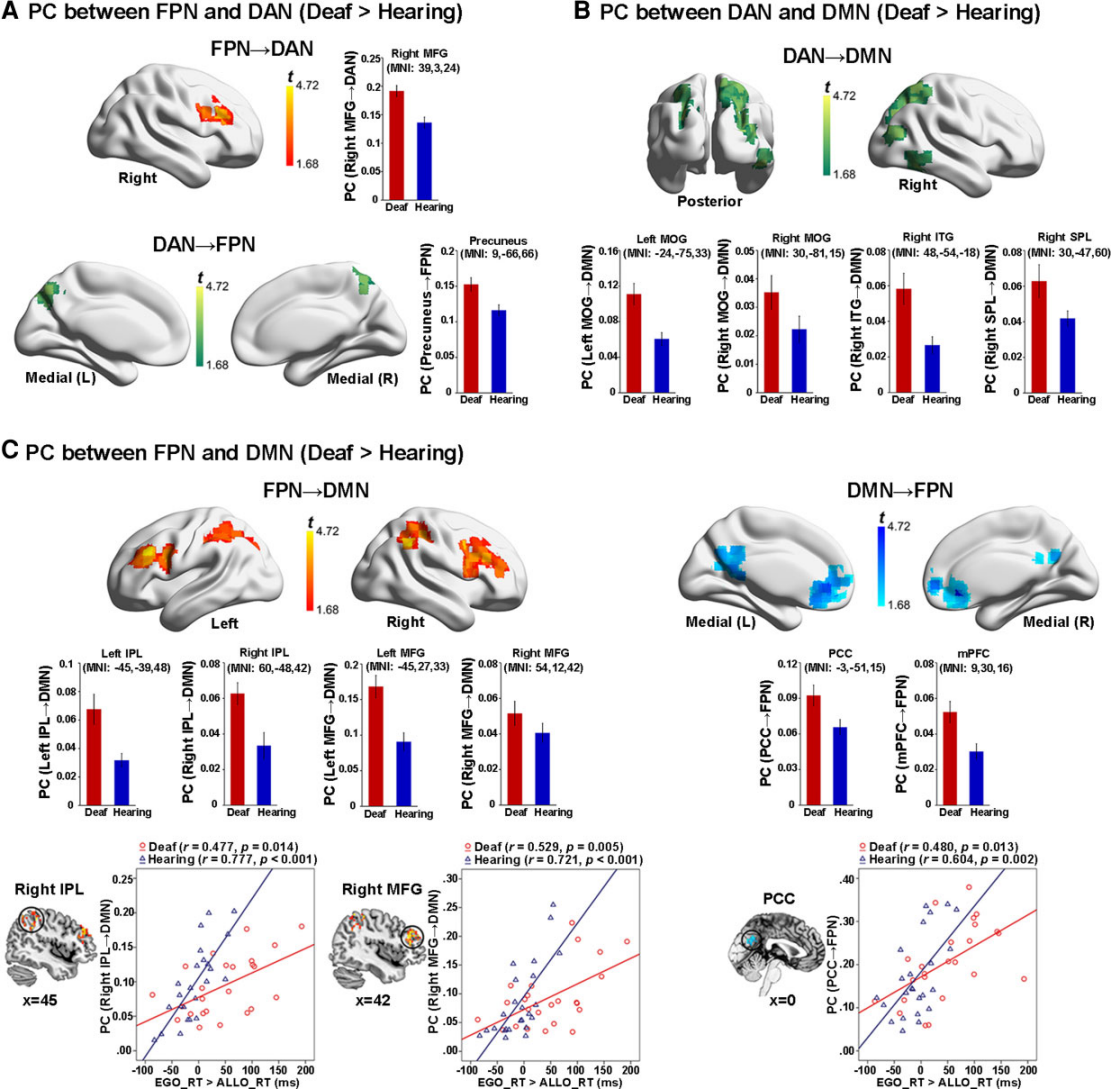
**Nodal-wise results of intrinsic neural activity during the resting state.** (**A**) Both the right MFG in the FPN (*top panel*) and the precuneus in the DAN (*bottom panel*) showed a significantly higher PC with the other task-critical network in the deaf than hearing group. (**B**) Extensive regions in the DAN showed significantly higher PCs to the DMN in the deaf than hearing group. (**C**) *Left panel:* the bilateral IPL and the bilateral MFG in the FPN showed significantly higher PCs to the DMN in the deaf than hearing group. Especially, the PCs in one right IPL and one right MFG cluster in the FPN significantly correlated with the egocentric performance in both groups. *Right panel*: the PCC and mPFC in the DMN showed significantly higher PCs to the FPN in the deaf than hearing group. Moreover, the mean PC value in a PCC cluster significantly correlated with the egocentric performance in both groups. For demonstration purposes, the mean PCs were extracted in the significantly activated regions and shown as a function of the two groups. No further statistical analysis was performed on the extracted PCs to avoid double-dipping. Error bars represent SEs.

The PC measures the extent to which a node connects to different modules other than its own. A node with a lower PC is more connected to the other nodes within their module, while a node with a higher PC is more connected to the nodes in the other modules. On the one hand, intrinsic neural activity in the two task-positive networks (DAN and FPN) was more significantly mutually coupled in the deaf than in the hearing brain during the resting state (Fig. 10A and Supplementary Table 6A). On the other hand, intrinsic neural activity in both the DAN (Fig. 10B and Supplementary Table 6B) and the FPN (Fig. 10C and Supplementary Table 6C) was more connected to the DMN in the deaf than in the hearing brain during the resting state. Moreover, the FPN–DMN PCs both in the right IPL and the right middle frontal gyrus (MFG) of the FPN (Fig. 10C, the two bottom left panels), and in the PCC of the DMN (Fig. 10C, the bottom right panel) were significantly positively correlated with egocentric performance, in both groups. The higher the intrinsic neural coupling between the FPN and the DMN in an individual participant, the slower the participant’s egocentric judgement. The correlation coefficient was generally higher in the hearing than in the deaf group. The above findings’ reproducibility was further proved by the graph theory analysis results based on the modularity partitions at a network density of 4% (Supplementary Fig. 3). Also, when the global signal was regressed out during the preprocessing of the resting-state fMRI, the above critical findings remained consistent (Supplementary Figs 4 and 5).

## Discussion

It has been well documented that auditory deprivation leads to both structural and functional reorganization in the deaf brain.^21,69–71^ Structurally, reduced white matter volume and density as well as reduced fractional anisotropy were observed in deaf children, adolescents and adults across the primary and secondary auditory cortex, and the superior temporal gyrus, which is involved in language processing.^72–78^ Also, early deprivation of auditory stimulation leads to reduced myelinization in the auditory cortex.^77,79^ Functionally, deaf individuals showed enhanced peripheral attention to motion, which involved the motion selective area in the MT/MST.^21,80–82^ Although the fact that the lack of early auditory input can lead to significant structural and functional alterations in the perception of the external world has been studied extensively in the literature (for reviews^21,22^), it remains poorly documented how the egocentric reference frame (i.e. the interface between the external world and the body), which is critical for all sensory-guided actions, is altered in the deaf population.

Auditory inputs play an essential role in the normal development of body-related processes. In typically developing individuals, auditory (and tactile) inputs interact with the motor system during posture and balance as well as during movement initiation.^23–26^ Accordingly, the lack of auditory experience in deaf people causes deficits in these body-related motor processes, as evidenced by poor procedural motor learning and postural stability^83–86^ as well as slower RTs and reduced movement speed.^31–35^ Using neuropsychological and fMRI experiments, we studied, whether early auditory deprivation affects egocentric processing in congenitally deaf participants. We addressed the following key questions: (i) Is it the fidelity of the visuospatial representations or the explicit sensorimotor transformation that is impaired by congenital deafness? (ii) What are the neural mechanisms underlying these behavioural alternations of the body-centred frame of reference in the deaf brain in terms of both the evoked neural activity during the task-state and the intrinsic neural activity during the resting state?

Behaviourally, the deaf participants were significantly slower than the hearing participants in explicit egocentric judgements (Fig. 1D). Furthermore, we provide direct evidence strongly suggesting that this slowed egocentric processing was due to a deficient body-centred sensorimotor transformation process rather than degraded visuospatial representations (Figs 2 and 3). In normal-hearing participants, both the egocentric and allocentric positions of target objects are automatically represented in the brain, irrespective of the task at hand.^10,87^ Specifically, when no explicit egocentric judgements (i.e. no explicit body-centred sensorimotor transformation) were required in the present study, the task-irrelevant egocentric positions of the behavioural targets were represented well in the deaf brain and caused similarly sized conflicts in the deaf and hearing group concerning either the allocentric positions in the allocentric task (Fig. 2B) or the side of the response hand in the HLB task (i.e. the typical Simon effect; Fig. 3A). These behavioural results support our hypothesis that the lack of early auditory input, together with the ‘parallel’ processing property of the intact visual input, biases the spatial reference frames in the deaf brain towards the outer end of the internal–external continuum (Fig. 1A) and results in impairments in the relatively internal body-centred frame of reference (and thus in egocentric processing), compared with the relatively external world-centred reference frame (Fig. 1D).

Auditory deprivation, even temporary, disturbs a variety of multisensory processes, such as audio-visual integration involving speech elements^88,89^ and audio-tactile integration during non-speech audio-tactile illusions.^90,91^ Two recent studies investigated the effects of temporary auditory deprivation^92^ and congenital deafness^27^ on the spatial localization of touch, in a crossed-arm temporal order judgement (TOJ) task.^93,94^ In the crossed-arm TOJ task, participants are asked to determine which of their two hands (left or right hand) received a tactile stimulus first, with their hands either uncrossed or crossed over the body midline. In the crossed-arm condition, a conflict is created between the anatomically anchored somatosensory reference frame, which is at the internal extreme end of the internal–external continuum, and the egocentric (body midline-centred) frame of reference, which is at the interface between the internal and the external ends^93–97^ (Fig. 1A). Note that the egocentric (body-centred) frame of reference is relatively external, compared with the somatosensory frame of reference. With the crossed hands, the right hand lies on the left side of the body midline, and the reverse for the left hand. If the spatial localization of touch relies exclusively on the internal somatosensory coordinates, the task performance should not be affected by the manipulation of the crossed versus uncrossed hands, i.e. irrespective of the egocentric position of the touch relative to the body midline. In contrast, if the egocentric body-centred reference frame interacts with the internal somatosensory reference frame to code the tactile stimulus location, the crossed hand manipulation should induce impairments in task performance. Accordingly, since congenitally blind people do not manifest any crossed-hand effects,^97^ it has been suggested that the lack of early visual input renders the touch localization in the early blind population mainly reliant on the internal somatosensory reference frame. More interestingly, compared with the normally developed hearing and sighted individuals, congenital deafness led to even larger impairments in the crossed-hand condition,^27^ and even temporary auditory deprivation in normal-hearing individuals led to impairments in the crossed-hand condition.^92^ Therefore, the lack of auditory input, even during temporal auditory deprivation, shifts the balance of spatial reference frames externally, leading to a heavier reliance on the relatively external body-centred frame of reference, compared with the relatively internal somatosensory frame of reference. In the present study, we further provided direct evidence that compared with the relatively external allocentric (world-centred) frame of reference, early deafness impairs the relatively internal body-centred frame of reference (Fig. 1D). Together with previous evidence, the present results support the hypothesis that the lack of auditory input shifts the balance of the spatial reference frames towards the relatively external extreme end, i.e. more dependence on the body-centred than somatosensory coordinates in the previous crossed-arm TOJ tasks,^27,92^ and more reliance on the allocentric world-centred than the egocentric body-centred coordinates in the present study (Fig. 1A).

At the neural level, the brain continually adjusts its architecture to meet the demands of the ever-changing interaction between goal-directed actions and environmental inputs. Successful completion of a cognitive task relies on two crucial brain network configurations.^98,99^ First, a coherent interaction among the task-relevant networks to secure the recruitment of necessary task-relevant resources, active representations of task goals and optimization of information flow.^100–105^ Second, a well-maintained DMN modularity, which is critical for the brain to focus attention on the current task and filter out task-irrelevant distractions.^100,102,105–109^ In the present study, successful egocentric judgements require an efficient information flow between the visuospatial representations generated in the DAN and the sensorimotor representations generated in the FPN. Accordingly, during the egocentric judgements in the hearing brain, on the one hand, the DAN significantly interacted with the FPN (Fig. 6B, the top panels), and on the other hand, the regions within the FPN remained coherently interactive (Fig. 6A, the top panel), to facilitate the sensorimotor transformation of the body-centred representations. Interestingly, similar to the hearing controls, the coherent interactions within and between the two task-critical networks (FPN and DAN) remained relatively intact during the egocentric task (Fig. 6A, the bottom left panel), and was even enhanced during the resting state (Fig. 10A), in the deaf group. Notably, despite the preserved coherent interaction between the task-relevant networks, egocentric processing was still impaired in the deaf brain. Therefore, the impairments in egocentric processing caused by congenital deafness are most likely attributed to an altered DMN modularity.

Accordingly, in the congenitally deaf participants, besides the intact interactions within and between the DAN and the FPN, (i) both of the two task-critical networks were also rewired to the DMN both during the egocentric judgement task (Fig. 6A, the bottom right panel; Fig. 6B, the bottom panels; Fig. 7A, the bottom panel) and at rest (Fig. 10B and C); and (ii) the intra-DMN connectivity was reduced at rest (Fig. 9A). Critically, the stronger the inter-network connectivity between the DMN and the FPN, the worse the egocentric performance (Fig. 7B; Fig. 9B, the right panel; Fig. 10C, the bottom panels). Therefore, the impaired explicit egocentric judgements in the deaf are associated with the decreased modularity of the DMN. This decreased modularity is expressed as a weaker intra-network connectivity among the DMN regions and a stronger inter-network connectivity between the DMN and the task-related networks FNP and DAN. The hyper-active cross-talking between the DMN and the task-critical networks supposedly prevents the DAN’s visuospatial representations from being effectively transformed into the sensorimotor representations in the FPN to sufficiently support the egocentric judgements. Consistent with the present results, it has been suggested that both the reduced pre-stimulus modularity of the DMN^102^ and the increased pre-stimulus connectivity between the task-critical sensory system and the DMN^100^ predict failures in detecting near-threshold sensory stimuli. Moreover, the higher anti-correlation between the DMN and task-critical networks, both before and after the actual presentation of the target stimuli, is associated with better task performance, in terms of both faster reactions and lower error rates.^106^ Therefore, good task performance necessitates a well-maintained DMN modularity and a breakdown of the DMN modularity limits/constraints the performance in (cognitive) tasks. Accordingly, both the weaker intra-DMN connectivity and the stronger inter-network connectivity between the DMN and other networks are associated with worse task performance.^109^ Together with these previous findings, the present results suggest that the breakdown of the DMN modularity in the deaf brain, especially during egocentric processing, impairs the information flow from the perceptual network (DAN) to the sensorimotor network (FPN), and thus slows down the body-centred sensorimotor transformations.

In addition to hearing loss, a potential contribution of vestibular dysfunction to the impaired egocentric reference in deaf participants cannot be ruled out. Although according to participants’ self-reports all the deaf participants in the present study experienced no balance problems, congenitally deaf people are generally unaware of concomitant vestibular damages.^110^ Moreover, hearing loss has a high comorbidity rate with vestibular impairments (up to 70%).^111–117^ Vestibular function has critical impacts on perception, motor and body-related processes.^110,118–122^ Therefore, vestibular deficits might explain some of the present results. As a matter of fact, a critical area in the left PPC showed abnormally enhanced neural activity during egocentric processing in the deaf group of the present study (Fig. 5A, the lower panel). Both experimental studies using animals and human studies showed that extensive parietal areas, including the PPC (area 7), parietal operculum and temporo-parietal junction, are involved in vestibular processing.^122–127^ Therefore, the additional involvement of the left PPC during egocentric processing in the deaf brain might constitute a compensatory mechanism for vestibular deficits, when an external object location must be precisely judged with reference to the middle line of one’s own body. Future studies will need to disentangle whether the impaired egocentric processing observed in congenitally deaf people is the result of early auditory deprivation or vestibular impairment.

Note that the deaf participants in the present study all had similar onsets and durations of their hearing loss and used comparable modes of communication, all of which impact neural reorganization and behavioural performance in deafness.^128^ In one of our previous studies, we showed the same pattern of impaired egocentric processing in another more heterogeneous group of deaf individuals, in which all the deaf participants became deaf before 1 year of age, but the aetiology of their deafness varied across participants, i.e. either congenital or acquired.^29^ Since egocentric processing was impaired in both the present study and in the study by Zhang *et al*.,^29^ it is unlikely that the observed phenomenon is confined to the congenitally deaf population. Since all the present adult deaf participants were profoundly deaf already at birth, and none of them had CIs, it seems that a long period of profound deafness is required to induce deficient egocentric processing. However, it remains an open question whether the duration and the degree of deafness have an impact here. Further studies, with proper experimental variations of the onset, duration and degree of deafness, should link these factors to egocentric processing deficits, and should also evaluate whether a critical period exists, during which auditory input is required for normal egocentric processing. Also, all the deaf participants in the present study used sign language as a means of communication. Compared with hearing non-signers, both deaf and hearing signers have been shown to be faster in speeded mental rotation of objects.^129^ Previous studies suggest that allocentric reference frame is more dominant than egocentric reference frame during mental rotation.^130,131^ Accordingly, it could be possible that the enhanced mental rotation abilities in signers are due to the larger weights assigned to allocentric than egocentric reference frame.^132^ Therefore, sign language acquisition is a potential contributing factor to the observed effects in the present study. Future studies, with hearing signers, deaf signers and hearing non-signers, will need to dissociate the effect of sign language acquisition versus deafness on spatial reference frames. In addition, the use of hearing aids has been proven to impact plasticity and performance in the deaf.^133^ In the present study, the use of hearing devices varied from ‘never used’, over ‘used in the past’ to ‘currently used’ across our deaf participants. Moreover, the extent, to which participants understand speech with the hearing aid, varied from poor to well. Due to the heterogeneity of our deaf group, in terms of these two factors (frequency of using hearing aids and speech perception), we cannot specify the effects of using hearing devices on the current findings. We also acknowledge that we did not assess the duration of using hearing aids, the hearing thresholds with amplification, the hearing aid data logging and the hearing aid adjustment parameters in the present studies. Future research will need to investigate the effect of these hearing aid characteristics on egocentric processing.

In sum, our findings unravel a critical cause (i.e. impaired body-centred sensorimotor transformation) of a variety of hitherto unexplained difficulties in sensory-guided movements of the deaf population in their daily life (for review^19^). Furthermore, our data demonstrate the optimal network configurations between the task-positive and task-negative neural networks underlying coherent body-centred sensorimotor transformations. Early auditory input deprivation impairs the sensorimotor transformation from visuospatial representations of the external objects to their underlying sensorimotor representations relative to the body and its effectors. The impaired body-centred sensorimotor transformation is associated with abnormally increased cross-talk between the task-positive FPN and the task-negative DMN in the congenitally deaf brain, in terms of both evoked neural activity during egocentric processing and intrinsic neural activity during rest.

## Supplementary Material

fcac148_Supplementary_Data

## Abbreviations

AG =: angular gyrus
DAN =: dorsal attention network
DMN =: default-mode network
FPN =: frontoparietal network
HLB =: high-level baseline
IPL =: inferior parietal lobe
ITG =: inferior temporal gyrus
MFG =: middle frontal gyrus
mPFC =: medial prefrontal cortex
MTG =: middle temporal gyrus
PCC =: posterior cingulate cortex
PPC =: posterior parietal cortex
SEs =: standard errors
