# Supplementary material for: Impaired body-centred sensorimotor transformations in congenitally deaf people
Source: Brain Commun. 2022 Jun 7;4(3):fcac148. doi: 10.1093/braincomms/fcac148 (PMC9240416; doi:10.1093/braincomms/fcac148)
Supplement: fcac148_Supplementary_Data [file fcac148_supplementary_data.docx]

Supplemental Material For:

Impaired Body-centered Sensorimotor Transformations in Congenitally Deaf People

Hui Li^*^, Li Song^*^, Pengfei Wang^*^, Peter H. Weiss, Gereon R. Fink, Xiaolin Zhou, and Qi Chen

*: the authors contributed equally to the present work.

**Detailed analyses of behavioral data**

**Impaired egocentric performance in the deaf group**

RTs of valid trials were submitted to a generalized linear mixed model (GLMM) with groups (deaf vs. hearing), type of tasks (ALLO, EGO, and HLB) and their interaction as the fixed factors, and participants as a random intercept. Results showed that there was a significant main effect of the type of tasks (*χ*^2^ = 10.02, *p* < 0.01), indicating the overall RTs in the egocentric task (emm = 6.42, CI = [6.38;6.47]) were significantly slower than both in the allocentric task (emm = 6.39, CI = [6.35;6.44]; *z* = 8.11, *p* < 0.001) and in the nonspatial HLB task (emm = 6.40, CI = [6.36;6.45]; *z* = 5.95, *p* < 0.001). However, the main effect of groups was not significant (*χ*^2^ = 0.48, *p* > 0.05). Most importantly, the interaction between groups and the type of tasks was significant (*χ*^2^ = 164.06, *p* < 0.001). Further analysis on simple effects suggested that the overall RTs in the egocentric task were significantly slower in the deaf group (emm = 6.47, CI = [6.41;6.54]) than in the hearing control group (emm = 6.37, CI = [6.31;6.44]; *z* = 2.23, *p* < 0.05), while there was no significant difference between the deaf and the hearing group, either in the allocentric task or in the non-spatial HLB task, both *ps* > 0.05. For the accuracy data, the main effect of groups was significant (*χ*^2^ = 5.92, *p* < 0.05), indicating more errors in the hearing (emm = 2.52, CI = [2.32;2.72]) than the deaf (emm = 2.76, CI = [2.56;2.96]). The main effect of the type of tasks was not significant (*p* > 0.05). The interaction between groups and the type of tasks was significant (*χ*^2^ = 13.20, *p* < 0.01), further analysis on simple effects suggested that there were more errors in the egocentric task (emm = 2.48, CI = [2.27;2.70]) than both in the allocentric task (emm = 2.95, CI = [2.72;3.18]; *z* = -5.26, *p* < 0.001) and in the non-spatial HLB task (emm = 2.85, CI = [2.63;3.08]; *z* = -4.28, *p* < 0.001) in the deaf group, but no difference was found in the hearing group (all *ps* > 0.05). For demonstration purposes, mean RT for each participant was displayed in Fig. 1D, and mean error rate (calculated as the percentage of the error trials, the missed trials, and the outlier trials) was displayed in Fig. 1E.

The above behavioral results thus suggested that the egocentric processing was indeed impaired in the deaf group. However, it remained to be further elucidated whether the fidelity of the visuospatial representations of the egocentric positions or the sensorimotor transformation process underlying the explicit egocentric judgments causes the egocentric deficits in the deaf (Fig. 2A). To answer this critical question, we further disentangled the effect of the fidelity of the visuospatial representations and the process of explicit egocentric judgment by knocking out the necessity of body-centered sensorimotor transformation, such as in the allocentric task and the HLB (nonspatial luminance discrimination) task (Figs. 2B and 3A). If early deafness causes deficits specifically in the explicit body-centered egocentric judgments (i.e., sensorimotor transformation), without tampering with the fidelity of the visuospatial representations of the egocentric positions, we predicted that when the explicit egocentric judgments were not required in the allocentric judgment task and the HLB task, the task-irrelevant (but intact) visuospatial representations of the egocentric positions should cause comparable spatial conflicts between the deaf and the hearing group, either with the task-relevant allocentric positions in the allocentric task or with the side of the response hand in the HLB task (Fig. 2A). Please see the following detailed analysis of behavioral data in the spatial tasks and the nonspatial HLB task.

**The allocentric task: comparable spatial congruent effect caused by the task-irrelevant egocentric positions between the two groups**

To further investigate whether the fidelity of the visuospatial representations of the egocentric positions or the body-centered sensorimotor transformation was impaired by early deafness, we (1) manipulated the necessity of the body-centered transformation in the allocentric (no) vs. egocentric (yes) task; and (2) used the spatial congruency effect from the task-irrelevant to the task-relevant frame of reference to tag the fidelity of the visuospatial representations of the egocentric position when the necessity of explicit egocentric judgments was knocked out in the allocentric task. The better the visuospatial representations of the egocentric positions were represented, the larger spatial conflict effects they should cause. Similarly, in the egocentric task, the larger the spatial congruency effect caused by the task-irrelevant allocentric positions, the better the task-irrelevant allocentric positions were represented. Therefore, to measure the fidelity of the visuospatial representations of the egocentric positions when the explicit demands on the egocentric body-centered sensorimotor transformation were knocked out in the allocentric task (or vice versa in the egocentric task), a 2 (between-subject factor: deaf vs. hearing) × 2 (within-subject factor, type of task: ALLO vs. EGO) × 2 (spatial congruency between allocentric and egocentric position: congruent vs. incongruent) three-factorial design was tested on the behavioral data in the two spatial tasks.

Since the bottom-up input was slightly different between the congruent and incongruent condition (Fig. 2B, the left panel), we first tested the spatial congruency effect in the nonspatial HLB task in the hearing and deaf groups, respectively, to show that the spatial congruency effect between the two reference frames was not caused by the difference in the stimulus input. A GLMM analysis was performed with gamma distribution (link = log) in R 4.1.2 (R core team 2021; lme4 package)^1^. The type of spatial congruency in the HLB task (congruent vs. incongruent) was modeled as the fixed factor, RT of each valid HLB trial was the dependent variable, and the random intercept effect structured by participants was also included. For the accuracy data, a GLMM with binomial distribution (link = logit) was used with type of response (correct vs. incorrect) specified as the dependent variable. The fixed and random factors were the same as the RT analysis. The main effect was obtained from the fitted GLMM by using the “car” package (type III)^2^. The RT results showed that the RTs were comparable between the congruent and incongruent condition in the HLB task, in both the deaf group (*χ*^2^ = 0.65, *p* > 0.05), and the hearing group (*χ*^2^ = 2.30, *p* > 0.05). Similarly, the accuracy was comparable between the congruent and incongruent condition in the HLB task, in both the deaf group (*χ*^2^ = 0.11, *p* > 0.05) and the hearing group (*χ*^2^ = 3.08, *p* > 0.05). For demonstration purposes, mean RT (the right top panel) and mean error rate (the right bottom panel) for each participant in the non-spatial task were displayed in Fig. 2B and Supplementary Fig. 1. Therefore, the spatial congruency effect between the two spatial reference frames does not occur when neither frame of reference was task-relevant in the nonspatial HLB task, and thus is not simply induced by the slight difference in the bottom-up stimulus input.

Subsequently, RTs of valid trials in two spatial tasks were submitted to a GLMM analysis with gamma distribution (link = log). Groups (deaf vs. hearing), type of tasks (ALLO and EGO), spatial congruency between allocentric and egocentric position (congruent vs. incongruent), and interactions between any two variables were modeled as fixed factors, RT of each valid trial was the dependent variable, and the random intercept effect structured by participants was also included. For the accuracy data, a GLMM with binomial distribution (link = logit) was used with type of response (correct, incorrect) specified as the dependent variable. The fixed and random factors were the same as the RT analysis. The main effects and interactions were obtained from the fitted GLMM by using the “car” package (type III), and the simple effect was obtained by using the “emmeans” package^3^ with Bonferroni correction for multiple comparisons if there was a significant interaction. For the RTs, the main effect of groups was significant (*χ*^2^ = 5.64, *p* < 0.05), indicating that the deaf group (emm = 6.44, CI = [6.37;6.51]) was significantly slower than the hearing group (emm = 6.38, CI = [6.31;6.45]). The main effect of spatial congruency was significant (*χ*^2^ = 120.75, *p* < 0.001), indicating that RTs in the incongruent condition (emm = 6.45, CI = [6.40;6.50]) were significantly slower than in the congruent condition (emm = 6.37, CI = [6.32;6.42]). The two-way interaction between groups and type of tasks was significant (*χ*^2^ = 123.72, *p* < 0.001), further analysis on simple effects suggested that the RTs in the egocentric task were significantly slower in the deaf group (emm = 6.47, CI = [6.41;6.54]) than in the hearing control group (emm = 6.37, CI = [6.30;6.44]; *z* = 2.04, *p* < 0.05), while there was no significant difference between the deaf and the hearing group in the allocentric task (*p* > 0.05). Most importantly, the three-way interaction was significant (*χ*^2^ = 13.86, *p* < 0.001), further analysis on simple effects suggested that although the spatial congruency effects were significant in two groups, while the type of task interact with the spatial congruency effect differently between the deaf and hearing group. Specifically, for the hearing group, the spatial congruency effect was comparable in two spatial tasks (ALLO task: incongruency vs. congruency, *z* = 11.82, *p* < 0.001; EGO task: incongruency vs. congruency, *z* = 10.99, *p* < 0.001). However, for the deaf group, the spatial congruency effect was larger in the allocentric task (incongruency vs. congruency, *z* = 15.13, *p* < 0.001) than the egocentric task (incongruency vs. congruency, *z* = 6.52, *p* < 0.001). To further test this result, we calculated the spatial congruency effect of each spatial task for all participants. Given that the RTs distribution was positive skewed^4,5^, a log transformation was applied to the RTs, then we converted the transformed RTs into *Z*-value for each participant, and calculated the spatial congruency effect (individual mean *Z*-value of incongruent trials minus individual mean *Z*-value of congruent trials) of each task. Finally, the planned paired *t*-test was performed to compare whether the spatial congruency effect was different between the two tasks in the deaf and the hearing group, respectively. Results showed that the spatial congruency effect was significantly larger in the allocentric task than the egocentric task in the deaf (*t*_(25)_ = 3.11, *p* < 0.01), but no difference was found in the hearing (*t*_(23)_ = 0.51, *p* > 0.05). For demonstration purposes, mean RT for each participant in the allocentric task was displayed in Fig. 2B, the right top panel and Supplementary Fig. 1.

Taken together, only in the deaf group, the task-irrelevant egocentric positions caused significantly larger spatial conflicts to the allocentric judgment than vice versa, while the spatial congruency effect was comparable in the two spatial tasks in the hearing group. These results further suggested that the visuospatial representations of the egocentric positions were well maintained in the deaf brain when the necessity of body-centered sensorimotor transformation was knocked out in the allocentric task. However, the more demanding explicit egocentric judgment in the deaf than hearing group rendered less spatial conflicts from the task-irrelevant allocentric positions since it has been well documented that the more difficult a main experimental task, the less distractions from the task-irrelevant distractors^6–8^.

For the accuracy data, the main effect of spatial congruency was significant (*χ*^2^ = 89.00, *p* < 0.001), indicating more errors in the incongruent condition (emm = 2.20, CI = [2.05;2.36]) than in the congruent condition (emm = 3.36, CI = [3.17;3.54]). The interaction between groups and the type of tasks was significant (*χ*^2^ = 17.06, *p* < 0.001), further analysis on simple effects suggested that there were more errors in the egocentric task (emm = 2.57, CI = [2.34;2.80]) than in the allocentric task (emm = 3.20, CI = [2.94;3.46]; *z* = -5.97, *p* < 0.001) in the deaf group, while no difference was found in the hearing group (*p* > 0.05). In addition, the three-way interaction was significant (*χ*^2^ = 9.92, *p* < 0.01), further analysis on simple effects suggested that there were more errors in the hearing (emm = 3.21, CI = [2.91;3.51]) than the deaf (emm = 3.95, CI = [3.59;4.31]; *z* = -3.10, *p* < 0.05) in the congruent condition of the allocentric task. None of the other effects reach statistical significance, all *ps* > 0.05. For demonstration purposes, mean error rate for each participant in the allocentric task was displayed in Fig. 2B, the right bottom panel and Supplementary Fig. 1.

**The nonspatial HLB task: comparable Simon effect based on the task-irrelevant egocentric positions between the two groups**

To provide further supporting evidence that the visuospatial representations of the egocentric positions are intact and that only the explicit body-centered sensorimotor transformation is impaired in the deaf brain, we analyzed the Simon effect based on the allocentric vs. egocentric positions in the nonspatial HLB task. The logic behind this analysis was as follows: since the explicit spatial task demands (both egocentric and allocentric) were completely knocked out in the nonspatial HLB task, the task-irrelevant spatial representations of the target, especially the egocentric positions, were hypothesized to cause comparable conflicts to the side of the response hand (i.e., the classical Simon effect) between the hearing and the deaf group (Figs. 2A and 3A). To test this hypothesis, RTs of valid trials in non-spatial HLB tasks were submitted to a GLMM analysis with gamma distribution (link = log). Groups (deaf vs. hearing), Simon effect based on the allocentric position (congruent vs. incongruent), Simon effect based on the egocentric position (congruent vs. incongruent), and interactions between any two variables were modeled as fixed factors, RT of each valid trial was the dependent variable, and the random intercept effect structured by participants was also included. The accuracy data (type of response: correct vs. incorrect) was submitted to a GLMM analysis with binomial distribution (link = logit). The fixed and random factors were the same as the RT analysis. The main effects and interactions were obtained from the fitted GLMM by using the “car” package (type III), and the simple effect was obtained by using the “emmeans” package with Bonferroni correction for multiple comparisons if there was a significant interaction.

For the RTs, the main effect of the allocentric congruency was significant (*χ*^2^ = 66.04, *p* < 0.001), indicating significantly slower RTs in the allocentric incongruent condition (emm = 6.44, CI = [6.39;6.49]) than in the allocentric congruent condition (emm = 6.37, CI = [6.32;6.41]), i.e., a significant Simon effect based on the allocentric locations. The main effect of the egocentric congruency was significant as well (*χ*^2^ = 23.75, *p* < 0.001), indicating significantly slower RTs in the egocentric incongruent condition (emm = 6.42, CI = [6.38;6.47]) than the egocentric congruent condition (emm = 6.39, CI = [6.34;6.43]), i.e., a significant Simon effect based on the egocentric locations. None of the other effects reached statistical significance, all *ps* > 0.05. For demonstration purposes, mean RT for each participant in the non-spatial task was displayed in Fig. 3B, the left panel. Thus, these results suggested that when no explicit spatial processing was required in the nonspatial luminance discrimination (i.e., HLB) task, both the allocentric and the egocentric positions were well represented in both groups and caused an equivalent Simon effect between the hearing and the deaf group.

For the accuracy data, the main effect of allocentric congruency was significant (*χ*^2^ = 9.15, *p* < 0.01), indicating more errors in the allocentric incongruent condition (emm = 2.37, CI = [2.19;2.56]) than in the allocentric congruent condition (emm = 3.28, CI = [3.06;3.50]), i.e., a significant Simon effect based on the allocentric locations. The main effect of groups was significant (*χ*^2^ = 10.44, *p* < 0.01), indicating more errors in the hearing (emm = 2.55, CI = [2.30;2.80]) than the deaf (emm = 3.10, CI = [2.84;3.36]). The two-way interaction between groups and allocentric congruency was significant (*χ*^2^ = 4.82, *p* < 0.05), further analysis on simple effects suggested that there were more errors in the hearing (emm = 2.90, CI = [2.61;3.19]) than the deaf (emm = 3.66, CI = [3.32;3.99]; *z* = 3.36, *p* < 0.001) at the allocentric congruent condition, while there was no significant difference between the deaf and the hearing group in the allocentric incongruent condition (*p* > 0.05). None of the other effects reached statistical significance, all *ps* > 0.05. For demonstration purposes, mean error rate for each participant in the nonspatial task was displayed in Fig. 3B, the right panel. Therefore, the accuracy data replicated the RT data, showing comparable Simon effect based on the allocentric and egocentric positions between the deaf and the hearing group.

**
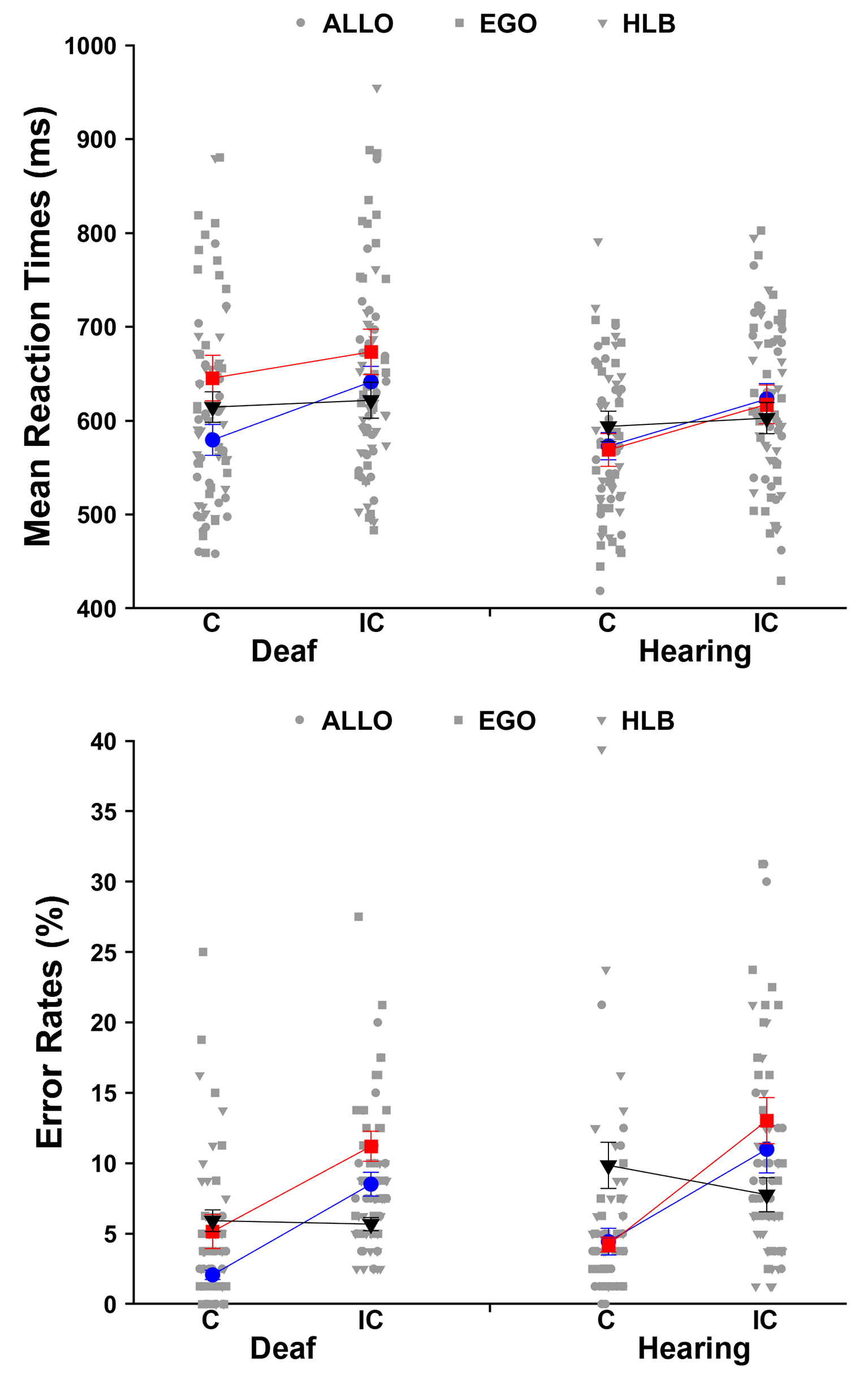
**

**Supplementary Figure 1. Spatial congruency effect between the allocentric and egocentric positions in the allocentric, egocentric, and HLB tasks. Error bars indicate standard errors (SE).**


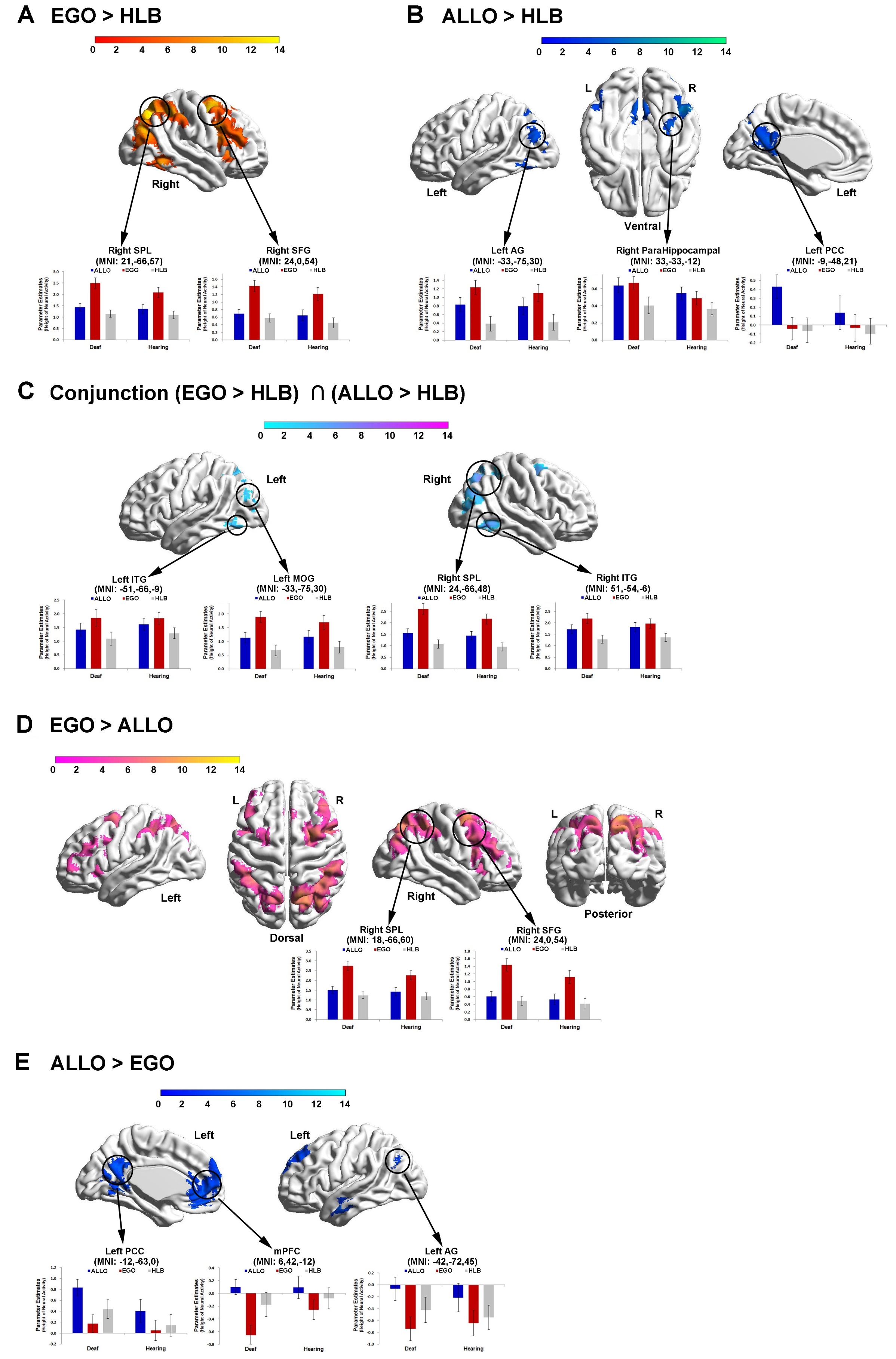


**Supplementary Figure 2. Mean beta values in the representative regions activated by the main effects of task (collapsed over the two groups).**

**
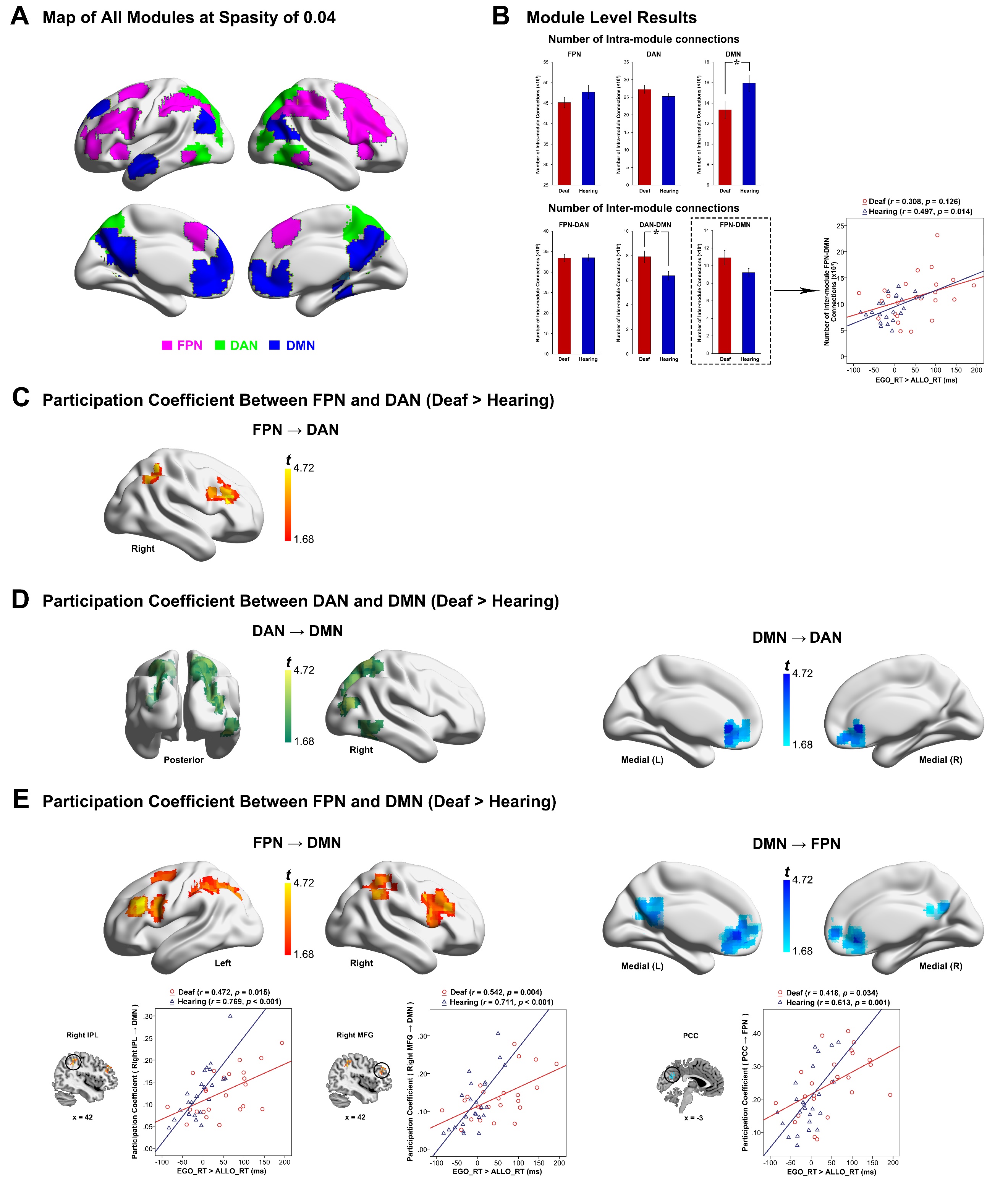
**

**Supplementary Figure 3. Results of graph theory analysis on the resting state fMRI data (without removal of global signal) at the sparsity of 0.04.**

**
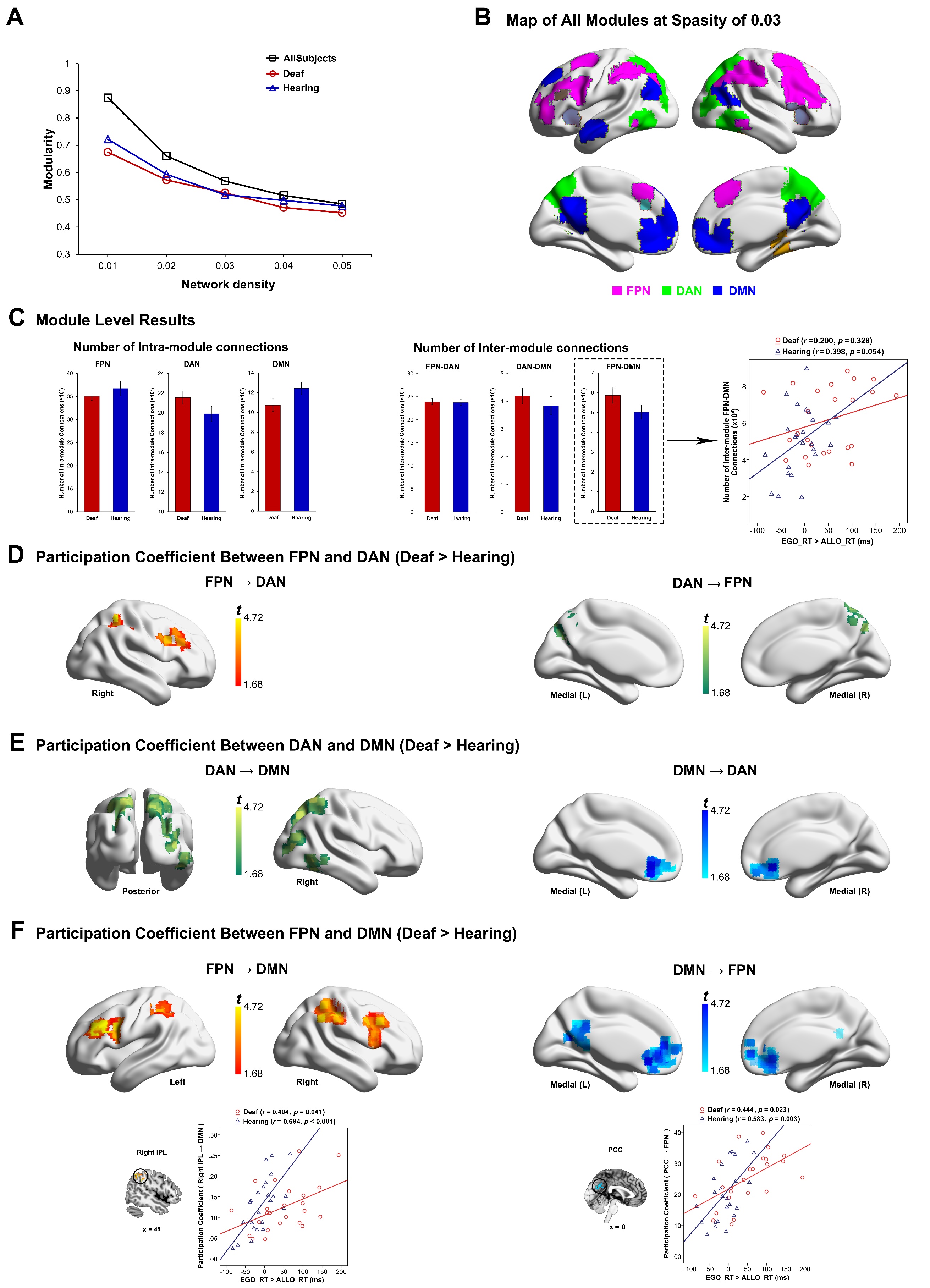
**

**Supplementary Figure 4. Results of graph theory analysis on the resting state fMRI data (with global signal being removed) at the sparsity of 0.03.**

**
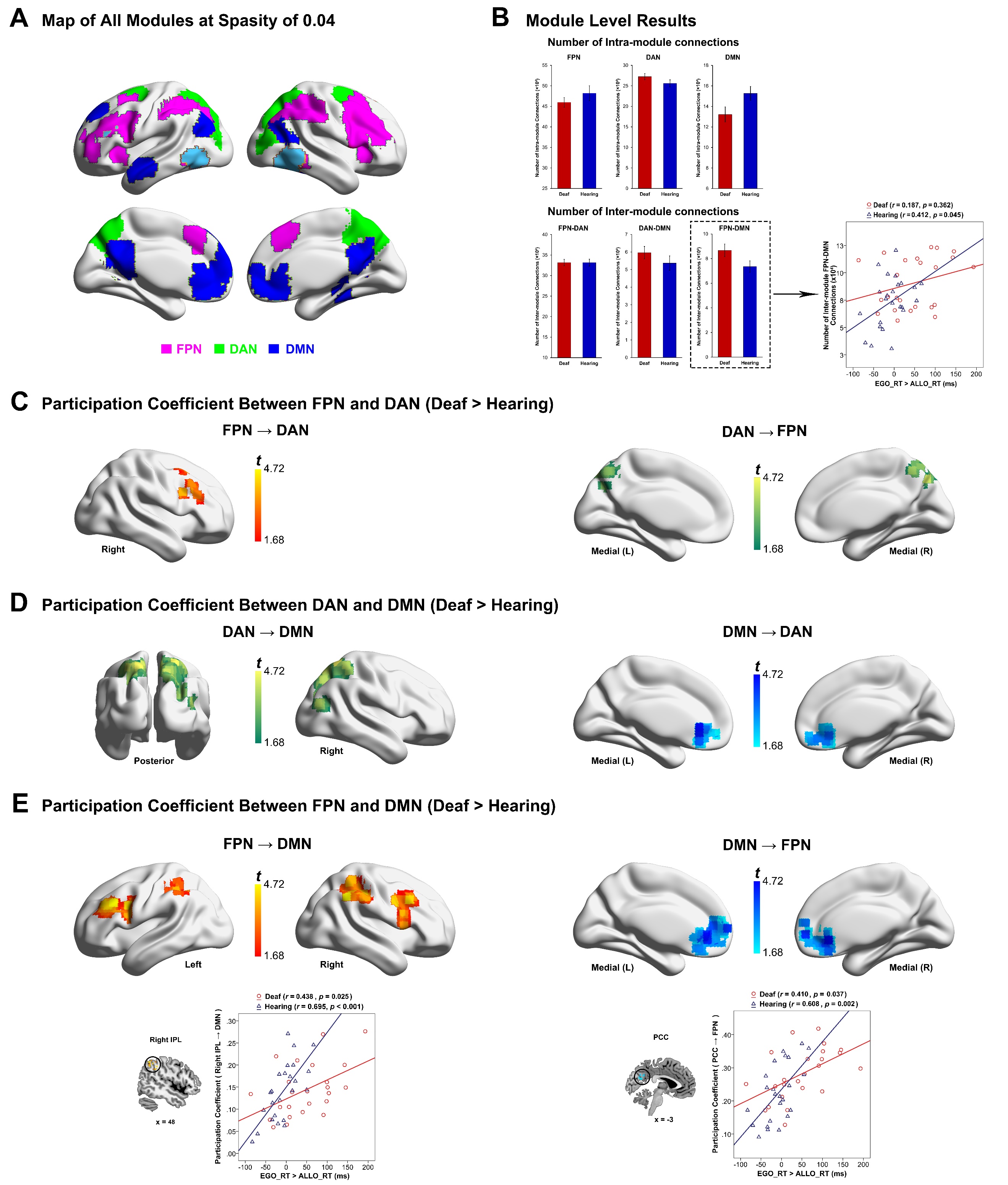
**

**Supplementary Figure 5. Results of graph theory analysis on the resting state fMRI data (with global signal being removed) at the sparsity of 0.04.**

**Supplementary Table 1.** Characteristics of the deaf participants.

| **Participant** | **Age (years)** | **Gender**  **(F/M)** | **Cause of deafness** | **Hearing loss**  **(left, right, dB)** | **Hearing aid use** | **How well the participant understands speech with hearing aid** | **Native language**  **(oral/sign)** | **Languages primarily used at the moment of the experiment** |
| --- | --- | --- | --- | --- | --- | --- | --- | --- |
| XLJ | 18 | M | Hereditary deafness | 100, 100 | Used in the past | Poorly | Sign | Sign |
| ZM | 20 | F | Disease/ drug side effects | 120, 100 | Uses currently | Well | Oral | Sign and oral |
| HQ | 21 | M | Hereditary deafness | 120, 125 | Used in the past | Poorly | Sign | Sign |
| WZH | 20 | F | Disease/ drug side effects | 137, 137 | Uses currently | Well | Oral | Sign and oral |
| RJP | 19 | M | Hereditary deafness | 100, 100 | Uses currently | Poorly | Sign | Sign |
| ZWQ | 19 | F | Disease/ drug side effects | 120, 125 | Uses currently | Well | Sign | Sign |
| QL | 20 | M | Disease/ drug side effects | 100, 100 | Uses currently | Poorly | Sign | Sign |
| GJH | 25 | M | Disease/ drug side effects | 120, 120 | Used in the past | Poorly | Sign | Sign |
| ZXN | 25 | M | Disease/ drug side effects | 90, 90 | Uses currently | Well | Sign | Sign |
| SJY | 24 | F | Hereditary deafness | 100, 120 | Uses currently | Poorly | Sign | Sign |
| GXH | 22 | M | Disease/ drug side effects | 90, 90 | Uses currently | Well | Sign | Sign |
| CL | 26 | F | Disease/ drug side effects | 102, 100 | Never used | - | Sign | Sign |
| WJ | 22 | M | Hereditary deafness | 95, 100 | Never used | - | Sign | Sign |
| XHC | 20 | F | Disease/ drug side effects | 90, 90 | Used in the past | Poorly | Oral | Sign |
| GGQ | 23 | M | Disease/ drug side effects | 120, 120 | Never used | - | Sign | Sign |
| ZZJ | 20 | F | Disease/ drug side effects | 105, 90 | Uses currently | Well | Oral | Sign and oral |
| WT | 20 | F | Hereditary deafness | 105, 105 | Never used | - | Sign | Sign |
| CMY | 23 | F | Hereditary deafness | 100, 90 | Used in the past | Poorly | Sign | Sign |
| BBX | 21 | M | Disease/ drug side effects | 90, 120 | Used in the past | Poorly | Oral | Sign |
| LK | 24 | F | Hereditary deafness | 120, 115 | Used in the past | Poorly | Sign | Sign |
| LMM | 21 | F | Disease/ drug side effects | 90, 100 | Never used | - | Sign | Sign |
| CXY | 22 | M | Disease/ drug side effects | 110, 110 | Uses currently | Poorly | Oral | Sign |
| ZW | 21 | F | Disease/ drug side effects | 100, 100 | Used in the past | Poorly | Sign | Sign |
| WXW | 23 | F | Disease/ drug side effects | 92, 108 | Used in the past | well | Oral | Sign and oral |
| YQQ | 21 | F | Disease/ drug side effects | 100, 100 | Uses currently | well | Sign | Sign |
| LB | 20 | M | Disease/ drug side effects | 120, 120 | Used in the past | Moderately | Sign | Sign |

Information was reported by participants on the day of the experiment. Hearing loss was determined using a standard pure-tone audiometry.

**Supplementary Table 2.** Main effect of experimental tasks. (**A**) Brain regions activated by the egocentric task (EGO), as compared to the HLB task. (**B**) Brain regions activated by the allocentric (ALLO), as compared to the HLB task. (**C**) Brain regions commonly activated by both the egocentric and the allocentric task, as depicted by the conjunction analysis of the two contrasts “EGO > HLB” and “ALLO > HLB”. (**D**) Brain regions specifically activated by the egocentric task, as compared to the egocentric task, i.e., “ALLO > EGO”. (**E**) Brain regions specifically activated by the egocentric task, as compared to the allocentric task, i.e., “ALLO > EGO”.

| **Anatomical Region** | **Side** | **Cluster Peak (mm)** | ***t*-Value** | **Ke (voxels)** |
| --- | --- | --- | --- | --- |
| **(A) EGO > HLB** | | | | |
| SPL | R | 21, -66, 57 | 13.42 | 4306 |
| *SPL* | *L* | *-15, -66, 54* | *9.37* |  |
| *IPL* | *R* | *36, -48, 51* | *10.72* |  |
| *ITG* | *R* | *54, -51, -9* | *9.81* |  |
| *IPL* | *L* | *-39, -42, 42* | *9.76* |  |
| SFG | R | 24, 0, 54 | 12.54 | 1769 |
| PreCG | L | -48, 6, 30 | 6.65 | 600 |
| MFG | L | -24, 0, 57 | 9.20 | 326 |
| Cerebellum | L | -24, -63, -30 | 7.15 | 266 |
| SFG | R | 6, 27, 45 | 6.81 | 262 |
| ITG | L | -51, -57, -6 | 6.71 | 154 |
| Hippocampus | R | 18, -30, 6 | 5.24 | 107 |
| Insula | L | -30, 21, 0 | 5.87 | 104 |
| **(B)** **ALLO > HLB** | | | | |
| SPL | R | 24, -66, 48 | 6.91 | 643 |
| *MOG* | *R* | *30, -69, 30* | *6.75* |  |
| PCC | L | -9, -48, 21 | 5.41 | 599 |
| MOG | L | -33, -75, 30 | 4.80 | 343 |
| ITG | R | 51, -54, -6 | 7.23 | 210 |
| para-hippocampus | R | 33, -33, -12 | 6.59 | 166 |
| *Hippocampus* | *R* | *21, -33, 3* | *6.20* |  |
| SFG | R | 24, 0, 54 | 5.12 | 90 |
| ITG | L | -51, -66, -9 | 4.17 | 79 |
|  |  |  |  |  |
| **(C) Conjunction: (EGO > HLB)** ∩ **(ALLO > HLB)** | | | | |
| SPL | R | 24, -66, 48 | 6.91 | 614 |
| *MOG* | *R* | *30, -69, 30* | *6.75* |  |
| MOG | L | -33 -75, 30 | 4.80 | 272 |
| ITG | R | 51, -54, -6 | 7.23 | 194 |
| SFG | R | 24, 0, 57 | 5.43 | 90 |
| ITG | L | -51, -66, -9 | 4.17 | 67 |
| **(D) EGO > ALLO** | | | | |
| SPL | R | 18, -66, 60 | 9.76 | 3216 |
| *SPL* | *L* | *-15, -69, 54* | *7.01* |  |
| *IPL* | *R* | *39.-48,51* | *8.89* |  |
| *IPL* | *L* | *-15, -69, 54* | *7.01* |  |
| SFG | R | 24, 0, 54 | 10.00 | 1783 |
| *Insula* | *R* | *33, 21, 3* | *5.55* |  |
| MFG | L | -45, 30, 33 | 5.85 | 352 |
| PreCG | L | -45, 6, 21 | 7.07 | 256 |
| MFG | L | -24, 0, 60 | 6.90 | 232 |
| SFG | M | 0, 27, 48 | 5.66 | 200 |
| Cerebellum | L | -24, -63, -30 | 5.57 | 198 |
| Insula | L | -33, 21, -3 | 4.86 | 56 |
| **(E) ALLO > EGO** | | | | |
| mPFC | R | 6, 42, -12 | 5.60 | 905 |
| PCC | L | -12, -63, 0 | 5.79 | 537 |
| MTG | L | -60, -3, -18 | 5.72 | 129 |
| AG | L | -42, -72, 45 | 4.38 | 76 |

The coordinates (x, y, z) correspond to MNI coordinates. Displayed are the coordinates of the maximally activated voxel within a significant cluster and the coordinates of relevant local maxima within the cluster (in *Italics*). SPL: superior parietal lobe, IPL: inferior parietal lobe, SFG = superior frontal gyrus, PreCG: precentral gyrus, MFG: middle frontal gyrus, ITG: inferior temporal gyrus, MOG: middle occipital gyrus, PPC: posterior parietal cortex, PCC: posterior cingulate cortex, mPFC: medial prefrontal cortex, MTG: middle temporal gyrus, AG: angular gyrus.

**Supplementary Table 3.** Direct comparisons between the allocentric and the egocentric task for the deaf and hearing group, respectively. Brain regions, specifically activated by the allocentric task, as compared to the egocentric task, are shown for the hearing (**A**) and the deaf (**B**) group. On the other hand, brain regions specifically activated by the egocentric task, compared to the allocentric task, are shown for the hearing (**C**) and the deaf (**D**) group.

| **Anatomical Region** | **Side** | **Cluster Peak (mm)** | ***t*-Value** | **Ke (voxels)** |
| --- | --- | --- | --- | --- |
| **(A)** **Hearing group EGO > ALLO** | | | | |
| IPL | R | 36, -45, 51 | 7.03 | 1680 |
| *SPL* | *R* | *18, -66, 57* | *6.92* |  |
| SFG | R | 24, 3, 60 | 6.41 | 922 |
| *IFG* | *R* | *48, 6, 21* | *6.81* |  |
| IPL | L | -42, -45, 51 | 4.72 | 207 |
| MFG | L | -27, 12, 57 | 4.23 | 52 |
| **(B) Hearing group ALLO > EGO** | | | | |
| oPFC | R | 9, 45, -9 | 4.01 | 64 |
| **(C) Deaf group EGO > ALLO** | | | | |
| SPL | R | 18, -75,51 | 7.63 | 1388 |
| *IPL* | *R* | *36, -54,48* | *7.34* |  |
| SFG | R | 27, 3, 54 | 7.26 | 995 |
| IPL | L | -39, -48, 48 | 6.71 | 921 |
| IFG | L | -45, 6, 24 | 6.26 | 169 |
| MFG | L | -24, 0, 60 | 5.70 | 133 |
| SFG | L | -3, 27, 45 | 4.69 | 117 |
| **(D)Deaf group ALLO > EGO** | | | | |
| mPFC | R | 6, 54, 9 | 5.14 | 550 |
| PCC | M | 0, -54,30 | 5.30 | 352 |
| MTG | L | -57, -3, -21 | 5.03 | 91 |

The coordinates (x, y, z) correspond to MNI coordinates. Displayed are the coordinates of the maximally activated voxel within a significant cluster and the coordinates of relevant local maxima within the cluster (in *Italics*). IPL: inferior parietal lobe, SPL: superior parietal lobe, SFG = superior frontal gyrus, IFG = inferior frontal gyrus, MFG: middle frontal gyrus, oPFC: orbital prefrontal cortex, ITG: inferior temporal gyrus, PCC: posterior cingulate cortex, MTG: middle temporal gyrus.

**Supplementary Table 4.** Neural correlates involved in the two spatial reference frames, specifically in the deaf group, as compared to the hearing group. (**A**) Brain regions specifically activated by the egocentric task in the deaf group. (**B**) Brain regions specifically activated by the allocentric task or deactivated by the egocentric task in the deaf group.

| **Anatomical Region** | **Side** | **Cluster Peak (mm)** | ***t*-Value** | **Ke (voxels) (voxels)** |
| --- | --- | --- | --- | --- |
| **(A)** **Deaf (EGO > ALLO) exclusively masked by Hearing (EGO > ALLO)** | | | | |
| PPC | L | -24, -63, 54 | 5.22 | 102 |
| **(B) Deaf (ALLO > EGO) exclusively masked by Hearing (ALLO > EGO)** | | | | |
| mPFC | R | 6, 54, 9 | 5.14 | 177 |
| PCC | M | 0, -54, 30 | 5.30 | 177 |

The coordinates (x, y, z) correspond to MNI coordinates. Displayed are the coordinates of the maximally activated voxel within a significant cluster and the coordinates of relevant local maxima within the cluster (in *Italics*). PPC: posterior parietal cortex, PCC: posterior cingulate cortex, mPFC: medial prefrontal cortex.

**Supplementary Table 5.** Results of the PPI analyses with seed regions in the left PPC of the FPN, the bilateral inferior temporal gyrus (ITG) in the DAN, and the mPFC in the DMN, and with “EGO > ALLO” as the psychological factor.

| **Anatomical Region** | **Side** | **Cluster Peak (mm)** | ***t*-Value** | **Ke (voxels)** |
| --- | --- | --- | --- | --- |
| 1. **the left PPC in the FPN as the seed** | | | | |
| ***the hearing group*** | | | | |
| SPL* | R | 18, -66, 54 | 6.48 | 558 |
| *IPL* | *R* | *39, -48, 54* | *4.81* |  |
| SPL* | L | -15, -69, 57 | 5,85 | 250 |
| PreCG | R | 30, -3, 45 | 4.45 | 108 |
| ***the deaf group*** | | | | |
| mPFC* | L | -3, 51, 36 | 5.47 | 709 |
| SPL | R | 24, -72, 51 | 4.68 | 475 |
| *PCC** | *L* | *-3, -51,30* | *4.19* |  |
| IPL | R | 42, -39, 51 | 4.60 | 325 |
| AG* | L | -54, -66, 27 | 4.49 | 149 |
| 1. **The bilateral ITG in the DAN as the seeds** | | | | |
| ***the left ITG: the hearing group*** | | | | |
| IPL | L | -36, -42, 36 | 5.43 | 384 |
| *SPL* | *L* | *-24, -60, 51* | *4.83* |  |
| IPL | R | 36, -45, 51 | 4.81 | 331 |
| *SPL* | *R* | *21, -60, 45* | *4.79* |  |
| ***the left ITG: the deaf group*** | | | | |
| PCC | M | 0, -57, 21 | 4.95 | 101 |
| ***the right ITG: the hearing group*** | | | | |
| SPL | R | 21, -66, 57 | 6.55 | 114 |
| IPL | R | 39, -45, 54 | 6.17 | 114 |
| SPL | L | -21, -63, 60 | 5.29 | 60 |
| SFG | R | 30, 0, 60 | 5.43 | 56 |
| MOG | R | 39, -78, 6 | 5.41 | 36 |
| ***the right ITG: the deaf group*** | | | | |
| PCC | R | 3, -54, 18 | 5.98 | 164 |
| AG | R | 51, -54, 24 | 5.14 | 138 |
| AG | L | -42, -63, 42 | 4.96 | 105 |
| 1. **the mPFC in the DMN as the seed** | | | | |
| ***the hearing group*** | | | | |
| PCC | R | 3, -48, 30 | 5.84 | 709 |
| AG | R | 48, -60, 30 | 5.16 | 475 |
| mPFC | M | 0, 51, 0 | 5.72 | 325 |
| AG | L | -54, -66, 27 | 3.79 | 149 |
| ***the deaf group*** | | | | |
| IPL | L | -33, -48, 39 | 5.22 | 85 |
| 1. **‘EGO_RT > ALLO_RT’ Correlation in the hearing group** | | | | |
| IPL | L | -57, -39, 45 | 4.17 | 251 |
| IPL | R | 42, -33, 39 | 4.11 | 141 |

The coordinates (x, y, z) correspond to MNI coordinates. Displayed are the coordinates of the maximally activated voxel within a significant cluster and the coordinates of relevant local maxima within the cluster (in *Italics*). *: the activated regions based on the left PPC seed region also survived a more conservative threshold at p < .05, FWE correction for multiple comparisons at the cluster level with an underlying voxel level of p < .001, uncorrected. SPL: superior parietal lobe, IPL: inferior parietal lobe, PreCG: precentral gyrus, SFG = superior frontal gyrus, AG: angular gyrus, PCC: posterior cingulate cortex, MOG: middle occipital gyrus, oPFC: orbital prefrontal cortex.

**Supplementary Table 6.** PC results at the nodal level.

| **Anatomical Region** | **Side** | **Cluster Peak (mm)** | ***t*-Value** | **Ke (voxels)** |
| --- | --- | --- | --- | --- |
| 1. **Participation Coefficient Between FPN and DAN (Deaf > Hearing)** | | | | |
| **FPN → DAN** | | | | |
| MFG | R | 39, 3, 24 | 4.13 | 81 |
| **DAN → FPN** | | | | |
| Precuneus | R | 9, -66, 66 | 3.94 | 82 |
| 1. **Participation Coefficient Between DAN and DMN (Deaf > Hearing)** | | | | |
| **DAN → DMN** | | | | |
| MOG | L | -24, -75, 33 | 4.08 | 90 |
| MOG | R | 30, -81, 15 | 3.73 | 88 |
| ITG | R | 48, -54, -18 | 3.34 | 73 |
| SPL | R | 30, -47, 60 | 3.38 | 131 |
| 1. **Participation Coefficient Between FPN and DMN (Deaf > Hearing)** | | | | |
| **FPN → DMN** | | | | |
| IPL | L | -45, -39, 48 | 3.43 | 132 |
| IPL | R | 60, -48, 42 | 4.58 | 111 |
| MFG | L | -45, 27, 33 | 4.47 | 182 |
| MFG | R | 54, 12, 42 | 3.91 | 104 |
| ***‘EGO_RT > ALLO_RT’ Correlation*** | | | | |
| PostCG | R | 45, -30, 51 | 4.41 | 108 |
| *IPL* | *R* | *60, -39, 42* | *3.93* |  |
| MFG | R | 42, 36, 33 | 3.56 | 91 |
| **DMN → FPN** | | | | |
| PCC | L | -3, -51, 15 | 3.24 | 107 |
| mPFC | R | 9, 30, 16 | 4.72 | 148 |
| ***‘EGO_RT > ALLO_RT’ Correlation*** | | | | |
| PCC | M | 0, -60, 33 | 3.94 | 73 |

The coordinates (x, y, z) correspond to MNI coordinates. Displayed are the coordinates of the maximally activated voxel within a significant cluster and the coordinates of relevant local maxima within the cluster (in Italics). MFG: middle frontal gyrus, MOG: middle occipital gyrus, ITG: inferior temporal gyrus, SPL: superior parietal lobe, IPL: inferior parietal lobe, PosCG: postcentral gyrus, PCC: posterior cingulate cortex, mPFC: medial prefrontal cortex.

**References**

1. Bates D, Mächler M, Zurich E, Bolker BM, Walker SC. Fitting Linear Mixed-Effects Models Using lme4. *JSS J Stat Softw*. 2015;67. doi:10.18637/jss.v067.i01

2. Fox, John; Weisberg S. *An R Companion to Applied Regression*. 2nd ed. SAGE Publications; 2011.

3. Lenth, Russell; Singmann, Henrik; Love, Jonathon; Buerkner, Paul; Herve M. Emmeans: Estimated Marginal Means, aka Least-Squares Means. *R Packag version*. 2018;1(1):3.

4. Baayen RH, Milin P. Analyzing Reaction Times. *Int J Psychol Res*. 2010;3(2):12-28. doi:10.21500/20112084.807

5. Lo S, Andrews S. To transform or not to transform: using generalized linear mixed models to analyse reaction time data. *Front Psychol*. 2015;6. doi:10.3389/fpsyg.2015.01171

6. Forster S, Lavie N. Failures to Ignore Entirely Irrelevant Distractors: The Role of Load. *J Exp Psychol Appl*. 2008;14(1):73-83. doi:10.1037/1076-898X.14.1.73

7. Lavie N, Hirst A, De Fockert JW, Viding E. Load theory of selective attention and cognitive control. *J Exp Psychol Gen*. 2004;133(3):339-354. doi:10.1037/0096-3445.133.3.339

8. Lavie N, Tsal Y. Perceptual load as a major determinant of the locus of selection in visual attention. *Percept Psychophys*. 1994;56(2):183-197. doi:10.3758/BF03213897
